# Rapid imaging of lung cancer using a red fluorescent probe to detect dipeptidyl peptidase 4 and puromycin-sensitive aminopeptidase activities

**DOI:** 10.1038/s41598-022-12665-9

**Published:** 2022-06-01

**Authors:** Shun Kawashima, Daisuke Yoshida, Takafusa Yoshioka, Akira Ogasawara, Kyohhei Fujita, Masahiro Yanagiya, Masaaki Nagano, Chihiro Konoeda, Haruaki Hino, Kentaro Kitano, Masaaki Sato, Rumi Hino, Ryosuke Kojima, Toru Komatsu, Mako Kamiya, Yasuteru Urano, Jun Nakajima

**Affiliations:** 1 Laboratory of Chemical Biology and Molecular Imaging, Graduate School of Medicine, The University of Tokyo, Tokyo, Japan; 2 Department of Thoracic Surgery, Graduate School of Medicine, The University of Tokyo, Tokyo, Japan; 3 Graduate School of Pharmaceutical Sciences, The University of Tokyo, Tokyo, Japan; 4 Department of Sports and Health Science, Daito Bunka University, Saitama, Japan

**Keywords:** Chemical biology, Cancer imaging, Lung cancer

## Abstract

Rapid identification of lung-cancer micro-lesions is becoming increasingly important to improve the outcome of surgery by accurately defining the tumor/normal tissue margins and detecting tiny tumors, especially for patients with low lung function and early-stage cancer. The purpose of this study is to select and validate the best red fluorescent probe for rapid diagnosis of lung cancer by screening a library of 400 red fluorescent probes based on 2-methyl silicon rhodamine (2MeSiR) as the fluorescent scaffold, as well as to identify the target enzymes that activate the selected probe, and to confirm their expression in cancer cells. The selected probe, glutamine-alanine-2-methyl silicon rhodamine (QA-2MeSiR), showed 96.3% sensitivity and 85.2% specificity for visualization of lung cancer in surgically resected specimens within 10 min. In order to further reduce the background fluorescence while retaining the same side-chain structure, we modified QA-2MeSiR to obtain glutamine-alanine-2-methoxy silicon rhodamine (QA-2OMeSiR). This probe rapidly visualized even borderline lesions. Dipeptidyl peptidase 4 and puromycin-sensitive aminopeptidase were identified as enzymes mediating the cleavage and consequent fluorescence activation of QA-2OMeSiR, and it was confirmed that both enzymes are expressed in lung cancer. QA-2OMeSiR is a promising candidate for clinical application.

## Introduction

Among malignant neoplasms, primary lung cancer is the leading cause of death worldwide^1^. Aggressive surgical resection improves the prognosis, and is increasingly used for elderly patients, patients with low lung function associated with emphysema, and patients with incidentally detected tiny lung cancers. In these cases, the risk–benefit ratio has led to an increasing number of patients undergoing sublobar resections, such as wedge resection and segmentectomy, instead of classic lobectomy. However, sublobar resection may shorten the distance between the tumor and the resection margin, which increases the risk of local recurrence. Indeed, the local recurrence rate is reported to be about 6% after reduction resection of lung cancer^2^. It is difficult to detect microcarcinomas near the surgical margins with the naked eye, and it is not practical to submit all margins for rapid pathological diagnosis. Therefore, in order to reduce local recurrence and improve the prognosis, there is a need for auxiliary diagnostic methods that can rapidly visualize microcarcinomas.

Fluorescent probes have many medical applications, including rapid diagnosis of cancer cells and evaluation of pathological conditions such as infection and inflammation. For example, focusing on the fact that the activity of ɤ-glutamyl transpeptidase is increased in certain tumors, we developed ɤ-glutamyl hydroxymethyl rhodamine green (gGlu-HMRG) as a fluorescent probe that is activated by this enzyme^3^ (Supplemental Fig. 1A). This probe is expected to be applicable for rapid diagnosis and imaging-guided surgery of cancers such as breast cancer^4^ and liver cancer^5^. Furthermore, gGlu-HMRG showed 43.8% sensitivity and 84.9% specificity for detecting lung cancer in surgical specimens^6^, and its utility for non-small cell lung cancer was confirmed in a prognostic study^7^. However, the sensitivity and specificity of gGlu-HMRG are not sufficient for clinical application to detect lung cancer. We considered that the probe performance might be improved in two ways. First, a probe emitting red fluorescence is expected to provide increased sensitivity, because autofluorescence of lung tissue is minimal in this wavelength range. Secondly, a probe targeting plural target enzymes whose activity is increased in lung cancer is expected to show increased specificity.

Our laboratory has developed a series of red fluorescent probes (Supplemental Fig. 1B) based on the 2-methyl silicon rhodamine (2MeSiR) scaffold, which has an absorption maximum at 593 nm and a fluorescence maximum at 613 nm^8,9^. Notably, its absorption wavelength shifts to the shorter wavelength side when a peptide chain is introduced on the amino group of one of the xanthene rings via an amide bond. Thus, the resulting compound does not emit fluorescence upon excitation at around 590 nm, but after cleavage, the released fluorophore is efficiently excited at this wavelength and emits strong fluorescence. We also modified 2MeSiR to obtain 2-methoxy silicon rhodamine (2OMeSiR). In this case, the fluorescence of the peptide derivative is quenched by photo-induced electron transfer, and activated after cleavage of the peptide bond, affording a red fluorescent probe with a higher fluorescence activation ratio^10^ (Supplemental Fig. 1C).

**Figure 1:**
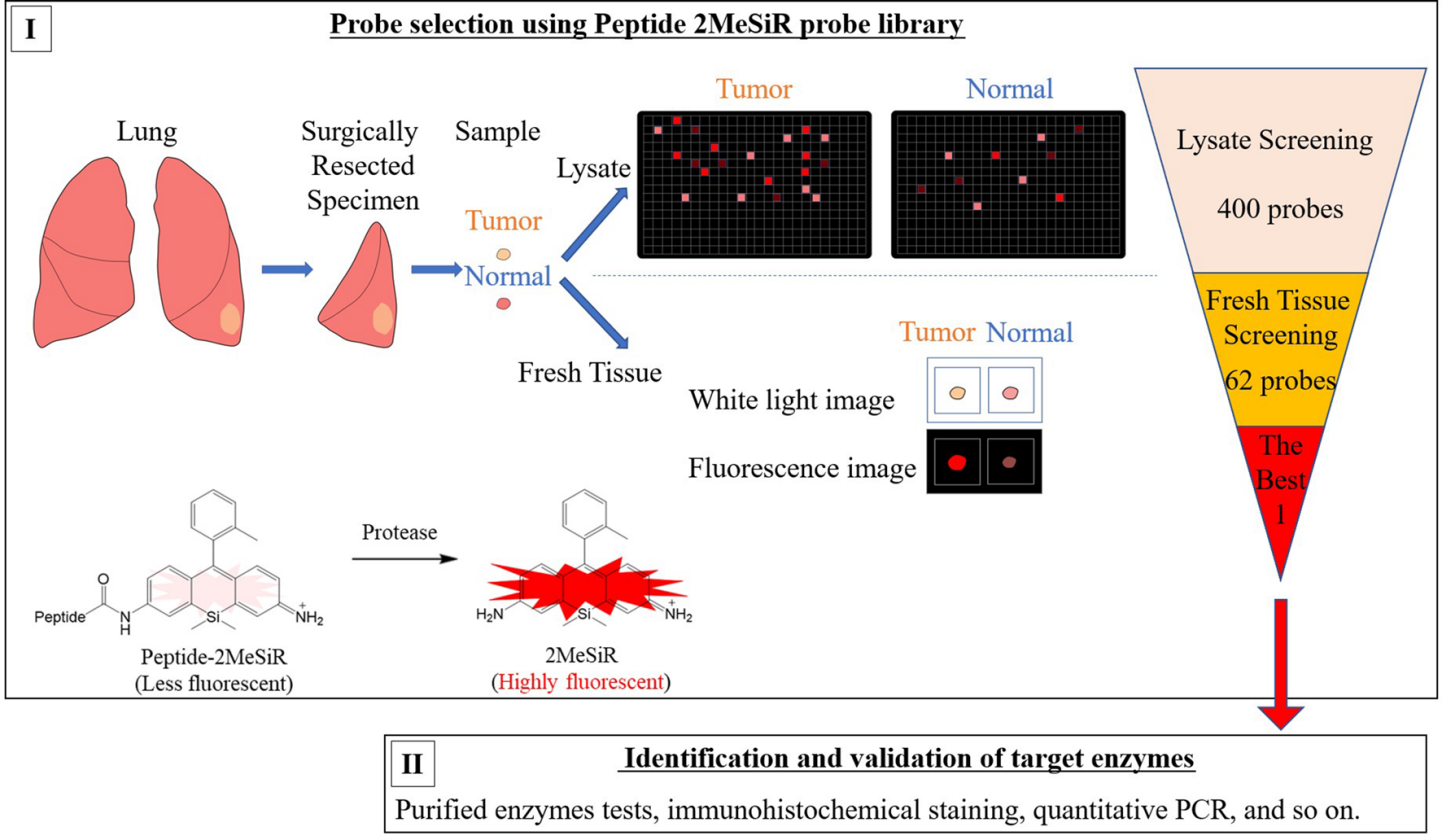
Schematic illustration of work-flow. (**I)** Lung cancer surgery specimens were used to screen a library of 400 2MeSiR-based probes. (**II)** After modification of the selected probe to improve the performance (see the text), the target enzymes were identified and validated using multiple methods.

Building on that work, the purpose of the present study is to select and validate the best red fluorescent probe for rapid diagnosis of lung cancer from a 400-probe library of 2MeSiR-based probes, as well as to identify the target enzymes that activate the probe, and to confirm their expression in cancer cells. We first screened the probes against lysates from lung cancer surgical specimens, and then tested the most effective candidates on fresh tissue specimens. Next, we modified the selected probe, QA-2MeSiR, to obtain glutamine-alanine-2-methoxy silicon rhodamine (QA-2OMeSiR), which has the same side chain structure as QA-2MeSiR but shows lower baseline fluorescence. The enzymes that activate QA-2OMeSiR fluorescence were identified and validated by several methods (Fig. 1).

## Results

### I-1. Screening using lung adenocarcinoma lysates

The peptide sequences of the probes and amino acid abbreviations are shown in Supplemental Tables 1 and 2. Lysates from 11 lung adenocarcinoma cases were used for initial screening. Among the 400 probes, 62 probes met the criteria of "elevated fluorescence of tumor lysate/normal lysate (TN ratio) ≥ 2.5” and “top 30% of elevated fluorescence of tumor lysate" in four or more cases; these are listed in Supplemental Table 3. An example of the change in fluorescence intensity over time is shown in Supplemental Fig. 2.

### I-2. Screening using fresh lung cancer specimens

**Figure 2:**
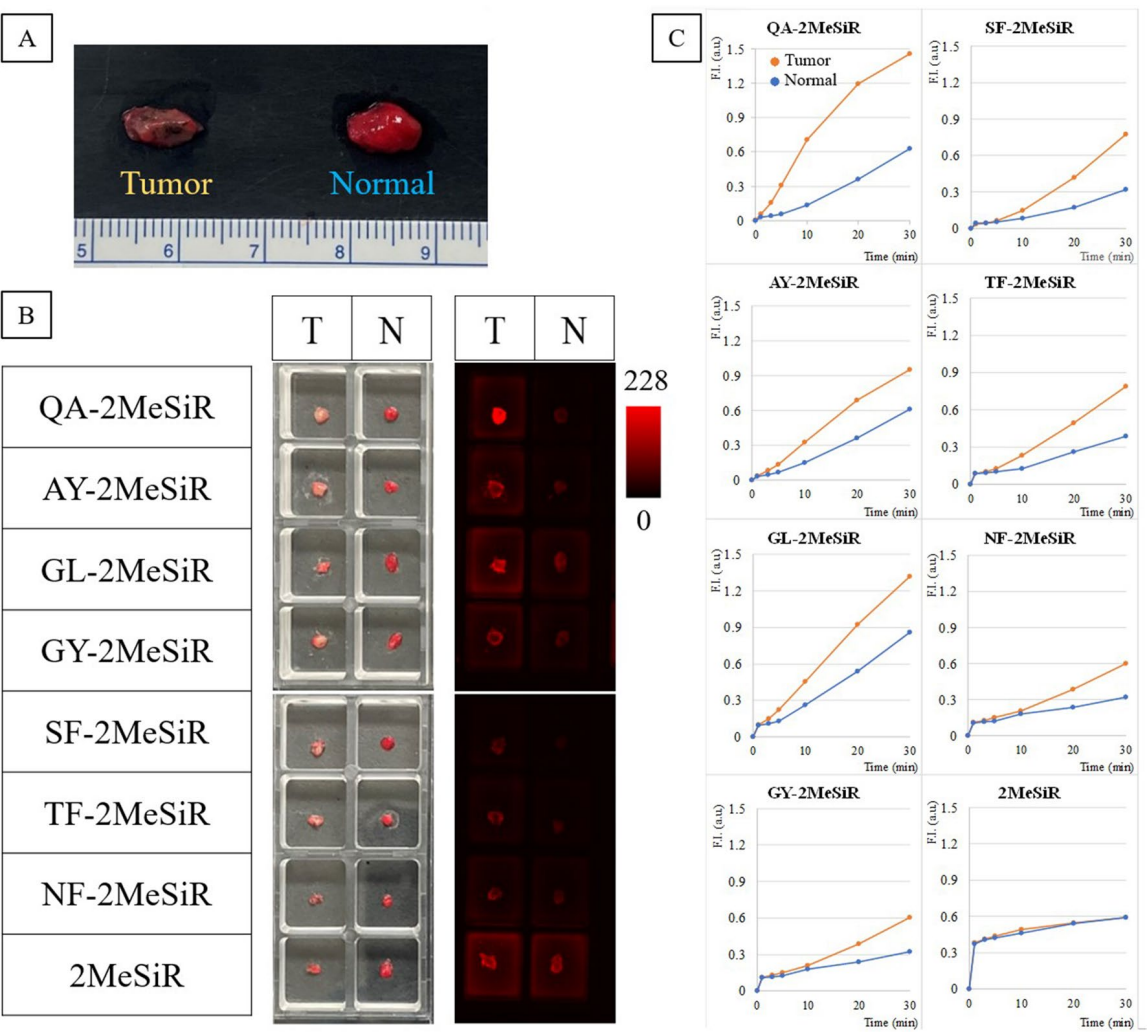
Imaging using fresh lung cancer specimens. (**A)** Photographs taken under ordinary fluorescent light. (**B)** Side-chain amino acids (single-letter abbreviations) of the fluorescent probes used in each well, image under general fluorescent light before probe application, and pseudo-color image taken under excitation light through a filter 10 min after probe addition. (**C)** Time course of fluorescence intensity of each probe. A large increase in tumor fluorescence and a small increase in normal tissue fluorescence are favorable.

A total of 23 fresh lung cancer specimens (16 adenocarcinoma, 5 squamous cell carcinoma, one atypical carcinoid, and one small cell carcinoma) were used to screen the 62 probes identified by lysate screening, together with 2MeSiR without any side chain. An example of the image and the change in fluorescence intensity over time is shown in Fig. 2. The results for the nine probes with AUC > 0.7 and 2MeSiR are shown in Supplemental Table 4. Among them, QA-2MeSiR showed the best AUC, in agreement with the result of adenocarcinoma lysate screening. Although there were only three squamous cell carcinoma cases, QA-2MeSiR showed good results with AUC = 1 and a mean tumor fluorescence increase/normal tissue fluorescence increase (hereafter referred to as T/N ratio) at 30 min after titration of 2.4. Among primary lung cancers, there were 11/18 cases with T/N ratio > 2 at 30 min, and the mean value of the T/N ratio was 2.2. Therefore, QA-2MeSiR was selected as the best candidate for lung cancer detection.

### I-3. QA-2MeSiR application to 27 primary lung cancer specimens

A total of 27 primary lung cancer specimens were pathologically proven to contain both tumor and normal tissue, including the 18 cases used for fresh specimen screening. The mean age of the patients was 67 years (26–83), 21 were male and 6 were female, and 21 cases had a smoking history. Tumor diameter averaged 29.8 mm (10–110), and histological types included 19 adenocarcinomas, five squamous cell carcinomas, one large cell neuroendocrine carcinoma, one atypical carcinoid, and one small cell carcinoma. The pathological stages were stage IA1 in one case, stage IA2 in eight cases, stage IA3 in five cases, stage IB in five cases, stage IIA in zero cases, stage IIB in four cases, stage IIIA in three cases, and stage IIIB in one case. Adenocarcinoma cases were numbered as #1–#19, squamous cell carcinoma cases as #20–24, the carcinoid case as #25, the large cell neuroendocrine carcinoma case as #26, and the small cell carcinoma case as #27.

**Figure 3:**
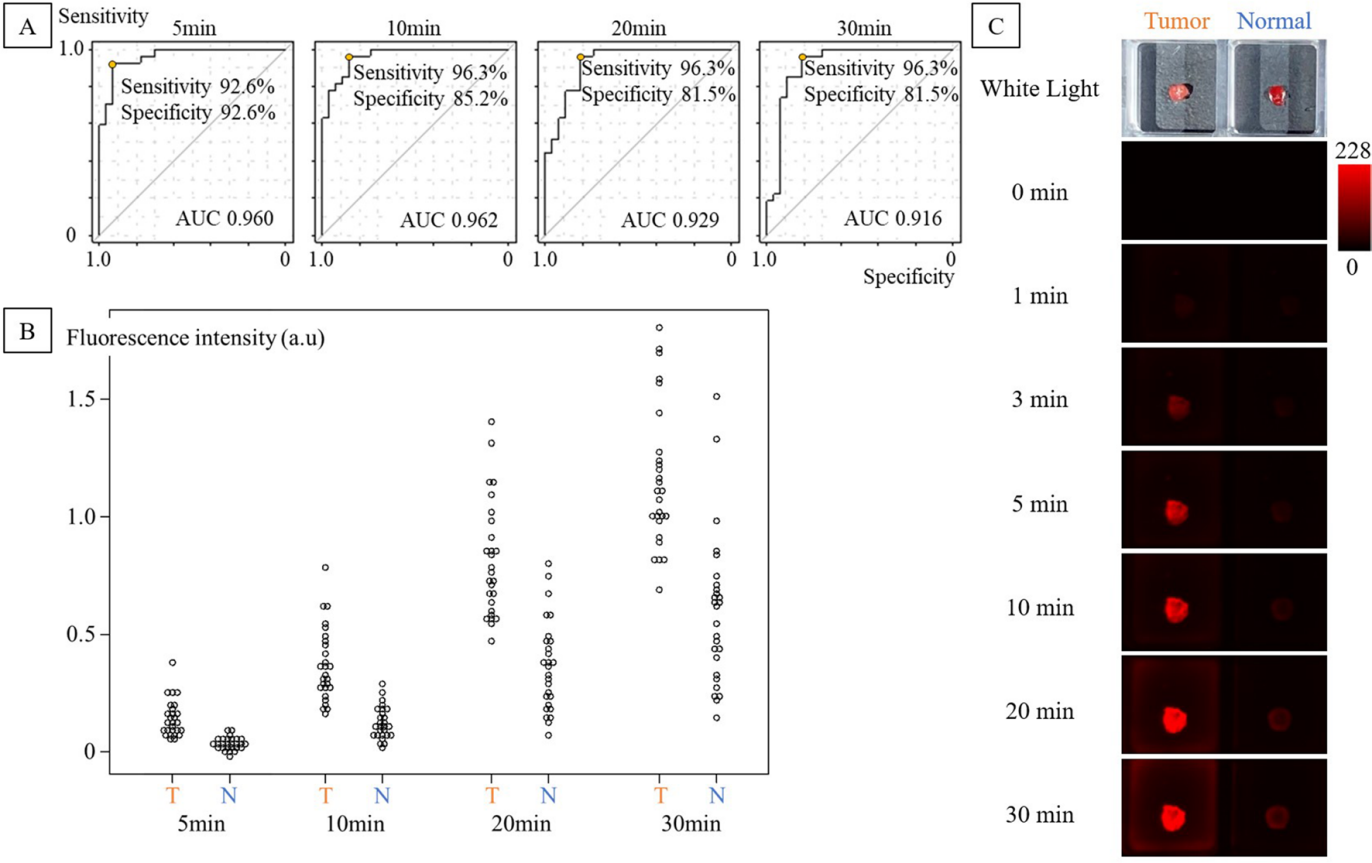
Imaging with QA-2MeSiR. (**A)** ROC curves of QA-2MeSiR at 5, 10, 20, and 30 min in 27 fresh tissue samples. QA-2MeSiR showed high sensitivity and specificity immediately after addition, and the best result was obtained at 10 min. (**B)** Dot charts of fluorescence intensity increase at 5, 10, 20, and 30 min. (**C)** Pseudo-color images showing fluorescence change after addition of QA-2MeSiR. An increase of fluorescence in the tumor can be recognized immediately after probe application.

Receiver operating characteristic (ROC) curves of QA-2MeSiR at 5, 10, 20, and 30 min are shown in Fig. 3, together with a dot chart showing the increase in fluorescence in tumor and normal tissues, and sequential pseudo-color images. Overall, the highest AUC (0.962) was observed at 10 min. The sensitivity was 96.3% and the specificity was 85.2%. The results are summarized in Supplemental Table 5. At 10 min, the average T/N ratio was 2.93, and T/N was ≥ 2 in 21/27 cases (78%). There were no significant differences by histological type, gender, or pathological stage within the measured range.

### I-4. In-vitro comparison of QA-2MeSiR and QA-2OMeSiR

In both fluorescence images measured by Maestro^®^ (CRi Inc., Woburn, MA) at 640 nm (Supplemental Fig. 3A) and digital camera images (iPhone6^®^ Apple, Cupertino, CA) (Supplemental Fig. 3C), which are considered to be similar to naked-eye observation, QA-2OMeSiR showed weaker fluorescence than QA-2MeSiR (Fig. 3C). On the other hand, there was no marked difference between 2OMeSiR and 2MeSiR in either case. The fluorescence intensity of 2OMeSiR was 52.8 times higher than that of QA-2OMeSiR, while that of 2MeSiR was 10.2 times higher than that of QA-2MeSiR (Supplemental Fig. 3D).

### I-5. Application of QA-2OMeSiR to marginal lung cancer lesions

**Figure 4:**
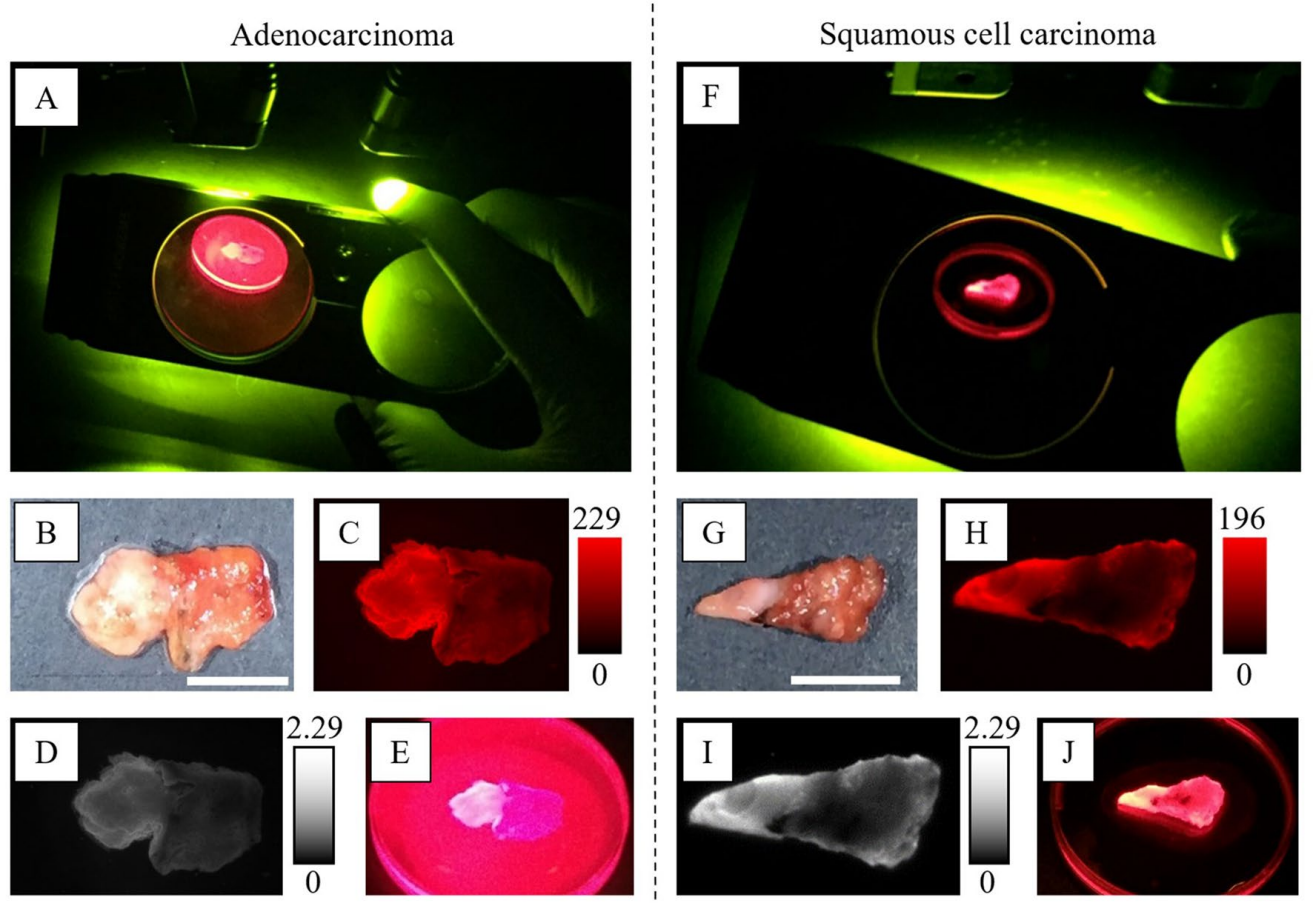
Borderline specimens measured with Maestro and a digital camera (**A–E**; adenocarcinoma (#13), **F–J**; squamous cell carcinoma (#24)). (**A,F)** QA-2OMeSiR was applied to a sample with a border between tumor and normal areas. The sample was photographed with an iPhone 6 under excitation after 30 min. (**B,G)** Image taken under white light before probe addition. The scale bar represents 1.0 cm. (**C,H)** Pseudo-color image (600–850 nm) of the specimen 10 min after probe addition. (**D,I)** Fluorescence image (640 nm) of a specimen at 10 min after probe addition. (**E,J)** Magnified image of a specimen taken at close range through a filter at 30 min after probe addition. Note: digital camera images have not been adjusted.

Examples of QA-2OMeSiR imaging of borderline lesions of adenocarcinoma and squamous cell carcinoma are shown in Fig. 4A–E and in Fig. 4F–J, respectively. HE staining confirmed the presence of tumor cells in the highly fluorescent areas and normal tissue in the weakly fluorescent areas. The image closest to that seen with the naked eye through the filter was the image taken by the digital camera, and this also showed a clear difference between tumor and normal tissues. Based on these results, the following studies were performed using QA-2OMeSiR.

### II-1. Selection of target enzyme candidates using diced electrophoresis gel assay

Diced electrophoresis gel (DEG) assay was performed to search for target enzymes, using QA-2OMeSiR and adenocarcinoma lysates #10 and #12. In the first assay, three peaks were observed (#10) (Supplemental Fig. 4A). These peaks were subjected to peptide mass fingerprinting (PMF) analysis as described below. DEG assay was also performed on lung adenocarcinoma sample lysate #12, and similar peaks were observed (Supplemental Fig. 4B). Next, DEG assay was performed with QA-2OMeSiR and squamous cell carcinoma lysate #21 (Supplemental Fig. 4C). In the squamous cell carcinoma lysate, there was no increase in fluorescence at the location of the major peak observed in the adenocarcinoma lysate, but there was a peak at the location corresponding to the weak peak observed in the adenocarcinoma lysate (③ in Supplemental Fig. 4A,B).

The PMF results afforded a list of candidate target enzymes (Supplemental Tables 6–8). The analysis of part ① in Supplemental Fig. 4 identified acylamino-acid-releasing enzyme (AARE) and dipeptidyl peptidase 4 (DPP4) as candidate target enzymes. The analysis of part ② yielded bleomycin hydrolase, calpain 1, and DPP4 as candidates, while part ③ afforded puromycin-sensitive aminopeptidase (PSA). Next, the putative involvement of these five enzymes was examined using inhibitors.

### II-2. Effect of inhibitors of target enzyme candidates on lung cancer lysate-induced QA-2OMeSiR fluorescence

**Figure 5:**
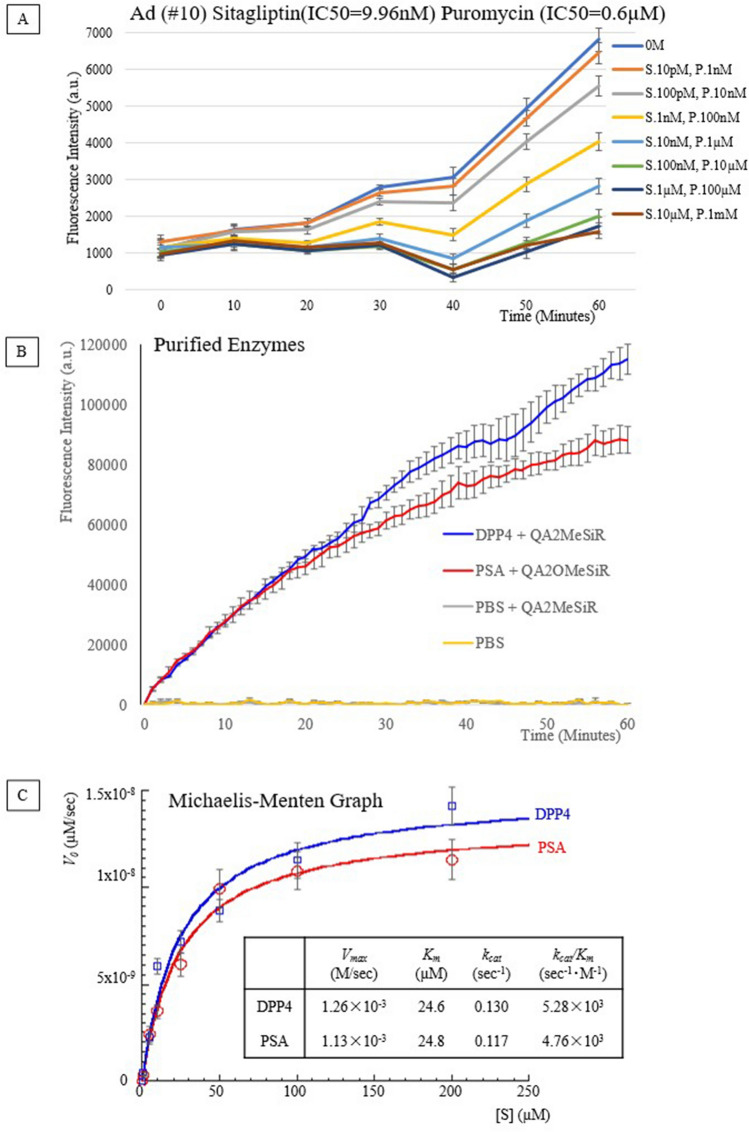
**(A**) Fluorescence of adenocarcinoma lysate and QA-2OMeSiR in the presence of various concentrations of sitagliptin and puromycin in combination. Puromycin at concentrations above 1 µM (above the IC_50_) suppressed the increase in fluorescence. When both sitagliptin and puromycin were used at concentrations around the IC50 values ("sitagliptin 10 nM, puromycin 1 µM"; light blue line) and above, the inhibitory effect was increased. Error bars indicate standard errors. Triplicate measurements were conducted using the same lysate and inhibitors. *S* sitagliptin, *P* puromycin. (**B)** Assay of purified enzymes and QA-2OMeSiR. The fluorescence intensity of purified DPP4 and PSA (0.114 µM) mixed with QA-2OMeSiR increases with time. (**C)** Plot of the initial velocities of the enzymatic reaction with DPP4 and PSA against concentration of QA-2OMeSiR, and the kinetic parameters obtained using the Michaelis–Menten equation, *V*_*0*_ = *V*_*max*_ × [S]/(*K*_*m*_ + [S]). The catalyst efficiency, *kcat/Km*, was about 1.1 times higher for DPP4 than for PSA. Error bars indicate standard errors. Triplicate measurements were conducted using enzymes with the same lot numbers.

We examined the effect of inhibitors of the candidate target enzymes on the fluorescence response of QA-2OMeSiR using lung adenocarcinoma specimen lysate #10 (Supplemental Figs. 5–9). Sitagliptin (DPP4 inhibitor), and puromycin (PSA inhibitor) were inhibitory, whereas AA74-1 (AARE inhibitor), E-6 (bleomycin hydrolase inhibitor), and calpain inhibitor II (calpain 1 inhibitor) were not, even at high concentrations. These results indicate that the target enzymes of QA-2OMeSiR are DPP4 and PSA. Since neither DPP4 nor PSA inhibitor alone fully blocked the fluorescence increase of QA-2OMeSiR, we performed an additional experiment using both sitagliptin and puromycin simultaneously (Fig. 5A). The combined inhibitors showed potent inhibition at concentrations above the IC50 (the results are shown in light blue in the figure for 10 nM sitagliptin, 1 µM puromycin and higher concentrations). Therefore, both DPP4 and PSA were considered to be target enzymes.

### II-3. Fluorescence activation of QA-2OMeSiR by purified DPP4 and PSA

Addition of either DPP4 (240 ng, MW = 105 kDa) or PSA (228 ng, MW = 100 kDa) to 1 µM QA-2OMeSiR probe solution resulted in a large increase in fluorescence (Fig. 5B). The kinetic parameters were evaluated by measuring the initial rate of the enzyme reaction with a plate reader (Fig. 5C). The enzyme concentration was standardized to 0.114 µM for both DPP4 and PSA. As shown in Fig. 5C, the Km values of QA-2OMeSiR with DPP4 and PSA were 24.6 µM and 24.8 µM, respectively. The catalytic reaction efficiency (*kcat/Km*) of DPP4 was about 1.1 times higher than that of PSA. In order to examine the enzymatic hydrolysis of QA-2OMeSiR by DPP4 and PSA, LC–MS analysis was performed using an ultra-performance liquid chromatography mass spectroscopy (UPLC–MS) system (Waters, Milford, MA). With both enzymes, an intermediate peak was observed corresponding to the cleavage of only Q, suggesting that a two-step reaction occurs at least in part, but the intermediate is quickly converted to 2OMeSiR (Supplemental Fig. 10).

### II-4. Effect of knockdown of DPP4 and PSA on lung cancer cell lines-induced QA-2OMeSiR fluorescence

**Figure 6:**
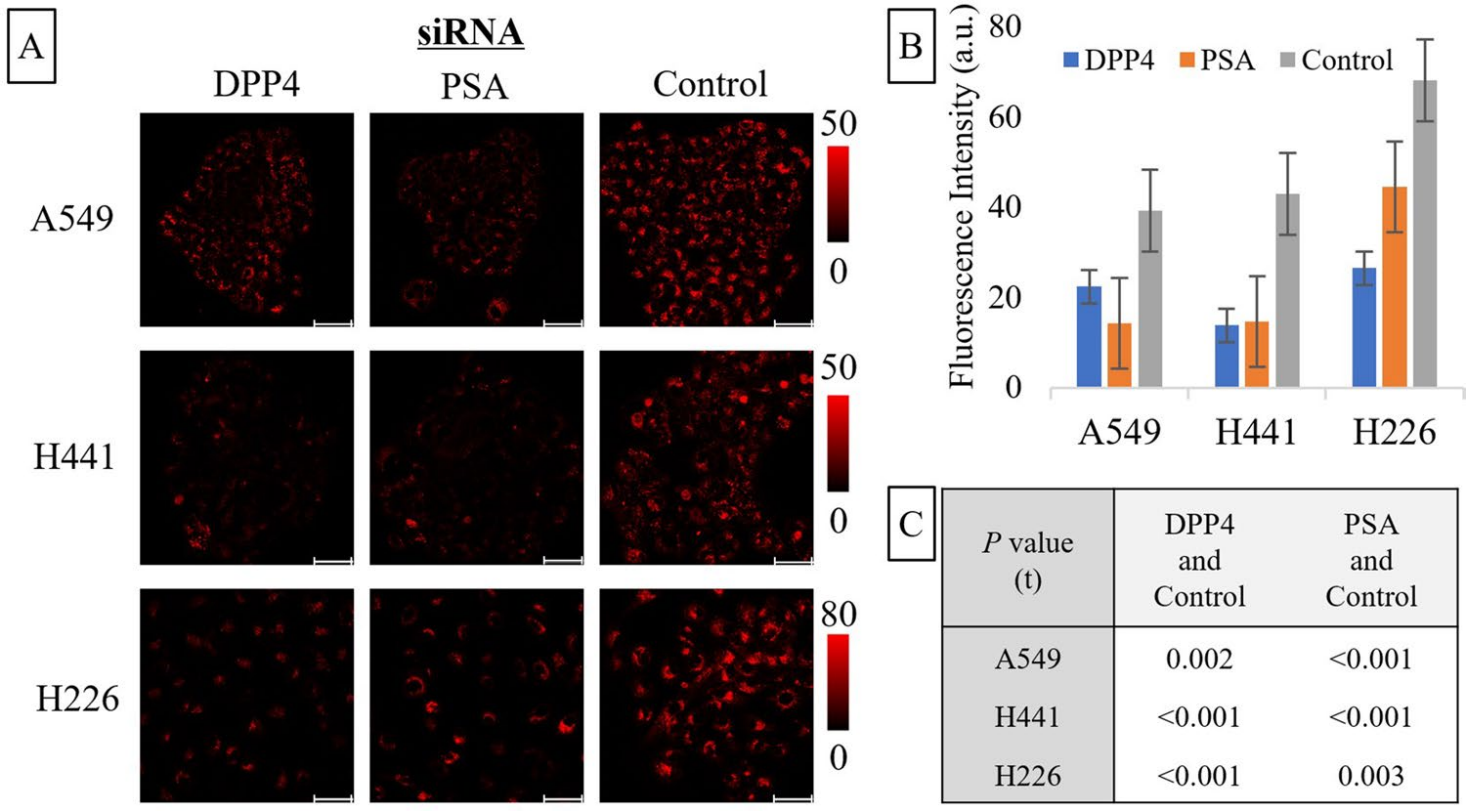
**(A)** Images taken 10 min after addition of QA-2OMeSiR to cell lines at 2 days after siRNA transfection. A549, H441, and H226 cells transfected with DPP4 siRNA or PSA siRNA show a suppressed increase of fluorescence compared to the Control siRNA-transfected cell lines. The scale bar represents 50 µm. (**B)** Increase in fluorescence intensity at 10 min after addition of QA-2OMeSiR, averaged over 15 cells. (**C)** Mean values of 15 measurements for each cell line and results of statistical comparison (t-test) of the effects of the siRNAs used.

Knockdown of DPP4 and PSA (also known as aminopeptidase puromycin sensitive; NPEPPS) was performed in three different lung cancer cell lines: A549 (adenocarcinoma), H441 (highly differentiated adenocarcinoma), and H226 (squamous cell carcinoma). Photographs of the reaction with QA-2OMeSiR at 2 days after knockdown are shown in Fig. 6A. Figure 6B,C show the mean values of 15 measurements for each cell line and the results of the t-test for differences between the siRNAs used. Knockdown of either DPP4 or PSA suppressed the response of QA-2OMeSiR. The fact that the suppression was only partial in each case supports the involvement of both enzymes in the reaction.

### II-5. Immunohistochemical staining

**Figure 7:**
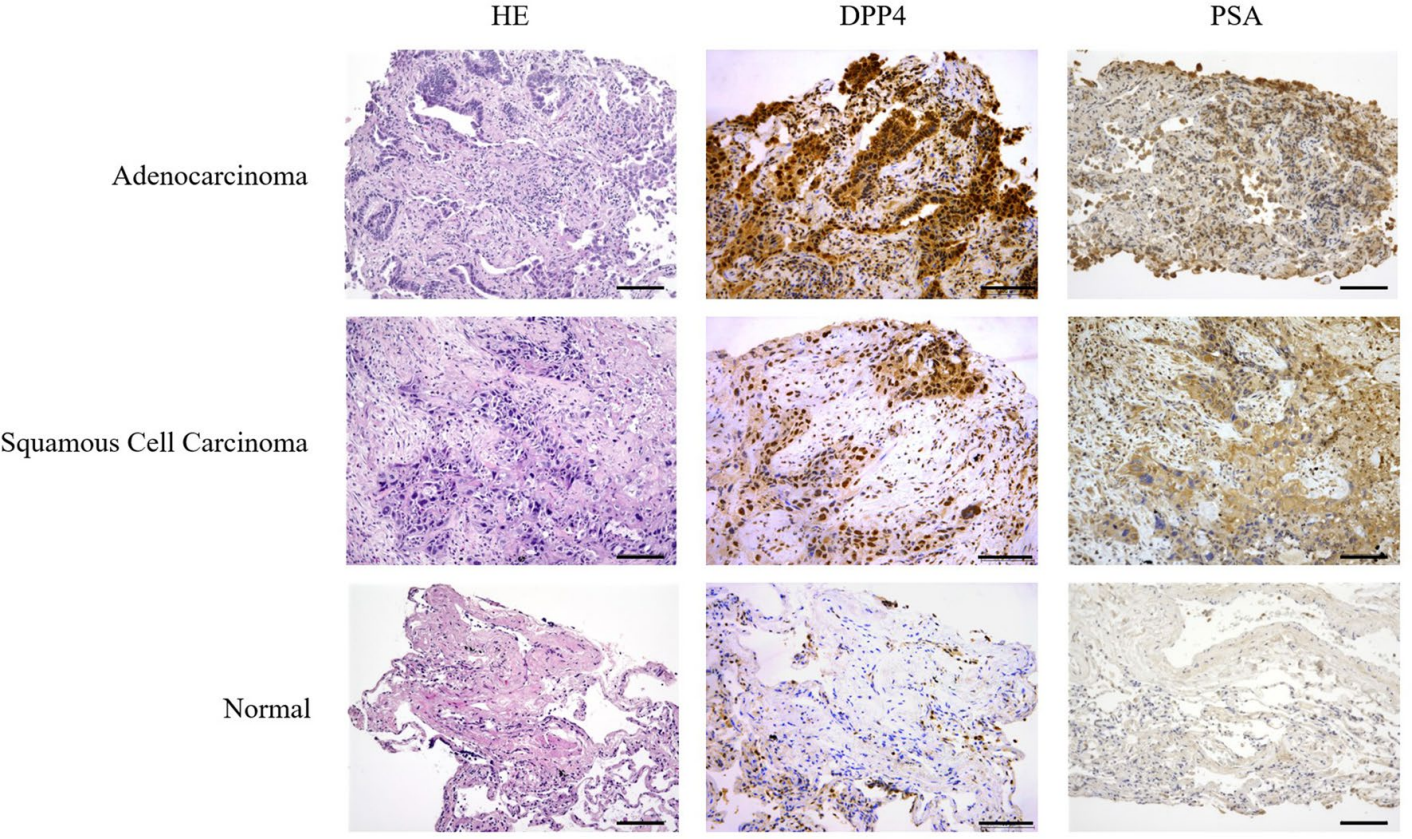
Pathological confirmation of the presence of DPP4 and PSA by HE staining and immunohistochemical staining of adenocarcinoma, squamous cell carcinoma and normal lung tissue. Both adenocarcinoma and squamous cell carcinoma are positive for DPP4 and PSA staining. In normal lung tissue, some macrophages and alveolar epithelium were positive for DPP4, but others were negative. PSA was negative in all normal tissues.

As shown in Fig. 7, both adenocarcinoma and squamous cell carcinoma showed positive immunostaining for DPP4. On the other hand, in normal lung tissue, some macrophages and alveolar epithelium were DPP4-positive, while others were negative. PSA staining was also positive in both adenocarcinoma and squamous cell carcinoma cells, while normal lung tissue was negative.

### II-6. Expression of DPP4 and PSA in lung cancer and normal tissue

**Figure 8:**
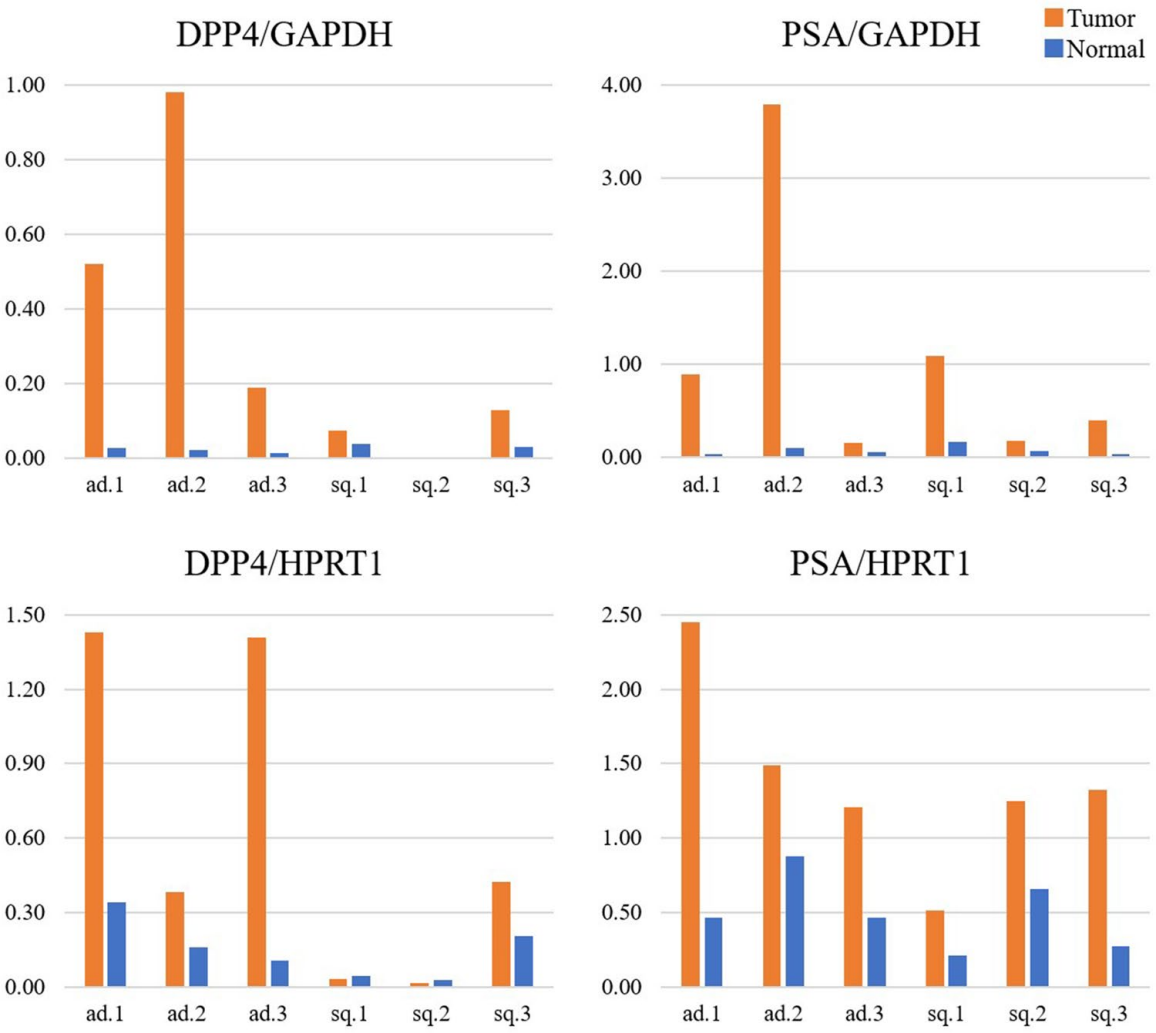
Quantitative PCR analysis of DPP4 and PSA expression in lung cancer. In adenocarcinoma, both DPP4 and PSA were more highly expressed in the tumor than in normal lung tissue (n = 3, #11, #14, #17). In squamous cell carcinoma, DPP4 was rarely observed in either normal or tumor tissues, but PSA was frequently observed in tumor tissues (n = 3, #22, #23, #24).

Whole RNA was extracted from lung cancer and normal lung tissues, and the expression levels of DPP4 and PSA were evaluated by means of quantitative polymerase chain reaction (qPCR) (Fig. 8). In adenocarcinoma, both DPP4 and PSA were more highly expressed in the tumor than in normal lung tissue (n = 3). In squamous cell carcinoma cases, DPP4 was weakly expressed in both normal and tumor tissues, whereas PSA was highly expressed in tumor tissues, but not in normal tissues (n = 3).

## Discussion

First, in connection with the probe screening, it should be noted that the results in the initial screen using frozen tissue lysates were not fully consistent with the results of the subsequent screen using fresh tissue samples, possibly due to loss of enzyme activity in the frozen samples. Therefore, it is important to confirm screening results in fresh specimens. We confirmed the absence of interference from tissue autofluorescence by using 2MeSiR (Supplemental Table 4).

In this study, QA-2MeSiR imaging of surgically resected fresh specimens showed a sensitivity of 96.3% and specificity of 85.2% at 10 min, representing a significant improvement over the results previously obtained with gGlu-HMRG^6^. Another probe, glutamic acid-proline-HMRG (EP-HMRG), showed a sensitivity of 96.9% and specificity of 85.7% for esophageal cancer^11^, and has also been applied to detect early-stage esophagogastric junction adenocarcinoma^12^ as well as head and neck cancer^13^. EP-HMRG is currently under clinical trial. In the present study, we did not find a correlation between fluorescence elevation and malignancy (histological type and progression) (Supplemental Table 5), but this may be due to the small number of cases and the lack of fresh specimens of early-stage cancer. Further studies will be needed to address this issue.

Two target enzymes, DPP4 and PSA, were identified according to the method of Komatsu et al.^14^. The finding that the fluorescence increase was almost completely suppressed by inhibitors of these two enzymes in the experiment with lung cancer tissue lysates also supports their involvement. In contrast, inhibitors of the other candidate enzymes had no effect in similar experiments, ruling out a contribution of these enzymes. This was confirmed by the use of siRNA-transfected cell lines, purified enzymes, immunostaining and qPCR. We found that the Km values of QA-2OMeSiR for DPP4 and PSA were 24.4 µM and 24.6 µM, respectively, and these values are similar to the reported Km value of 17.7 µM for EP-HMRG, which also targets DPP4^11^. Interestingly, not all of the samples expressed both DPP4 and PSA. For example, the qPCR results in Fig. 8 show that ad 1,2 and sq 3 express both DPP4 and PSA, but ad 3 expresses mainly DPP4, while Sq 2,3 expresses mainly PSA. This suggested that the lung cancers tissues examined here were heterogeneous.

DPP4 is a membrane-bound soluble protein that is widely distributed in the body^15^ and specifically cleaves the C-terminal peptide bond of the second alanine or proline from the N-terminal^16^. Physiologically, it inactivates incretin, an intestinal hormone that promotes insulin secretion, and is involved in immune regulation, signal transduction, and apoptosis^17^. It also plays a role in cancer progression and suppression, and is highly expressed in lung cancer cells^18^. PSA is a cytosolic alanyl aminopeptidase^19^, which may have a role in nervous system function^20,21^. Recent studies indicate that PSA inhibitors may be useful in the treatment of hematological malignant diseases^22^. Our siRNA, immunostaining and qPCR results indicated that not only DPP4 but also PSA is highly expressed in lung cancer cells. Thus, although neither DPP4 nor PSA is expressed specifically in cancer cells, the combination may be more effective than either enzyme alone to detect lung cancer cells. DPP4 is localized on the cell surface and PSA in the cytosol, and the reaction of QA-2OMeSiR with the enzymes is expected to occur at these locations after application of the probe (see Supplemental Fig. 11). It would be difficult to rationally design probes targeting both enzymes, and we consider that screening is an effective approach to find such multi-target probes.

We found that some normal lung tissues showed slight fluorescence elevation, which appeared to be associated with interstitial pneumonia or emphysema. Macrophages were immunostained for DPP4 (Fig. 7), and there are many reports showing that DPP4 has a role in the inflammatory process^23^. In addition, it has been reported that DPP4 is expressed in the alveolar epithelium during the development of fibrosis in interstitial pneumonia^24^. Thus, it is necessary to take into account the possibility that increased fluorescence may be observed in normal tissues in patients with such conditions.

In conclusion, our results indicate that the red fluorescent probe QA-2OMeSiR, which targets both DPP4 and PSA, is effective for the rapid detection of even borderline cancer lesions. This probe is a promising candidate for early clinical introduction. It may also have potential for application to detect metastatic lymph nodes and disseminated lesions. Furthermore, the screening strategy described here should be applicable to find probes for visualizing other types of malignant lesions that overexpress different enzymes.

## Methods

### Patients

Patients who underwent surgery for lung cancer at our institution between 2013 and 2021 were included in this study, which was approved by the Institutional Ethics Committee of the University of Tokyo ("Ethical Application 3900: Research on the Usefulness of Cancer-Specific Fluorescent Probes and the Development of New Probes in Lung Cancer"). All patients had given their informed consent to the use of their tissues for research purposes.

### Preparation of sample lysates

Tumor and normal samples from cryopreserved specimens of lung cancer patients were shredded with scissors, transferred to a Lysing Matrix D^®^ (MP Bio Japan, Tokyo, Japan), diluted with 1 mL of tissue protein extraction reagent (T-PER) (Thermo Fisher Scientific, Waltham MA), and homogenized. After centrifugation, the supernatant was collected, and protein quantification was performed.

### Measurement of fluorescence intensity with a plate reader

5 µL of lysate and 15 µL of probe solution (final concentration 1 µM) were added to a black plate (Corning 4511^®^, Corning, NY). Phosphate-buffered saline (PBS) ( +) with 1 mg/mL surfactant (CHAPS, Tocris Bioscience, Inc., Bristol, United Kingdom) was used to dilute probes. Measurements were taken every 2 min from 0 to 60 min with an EnVision^®^ (PerkinElmer Inc., Waltham, MA), and the difference between the 60-min value and the minimum value was taken as the fluorescence increase for each probe. Filter settings were BODIPY TMP FP (531/25 nm) for excitation and Cy5 (620/10 nm) for emission. All subsequent EnVision^®^ measurements were performed with the same filter settings.

### Selection criteria in lysate screening

Selection criteria were as follows: (1) fluorescence increase of tumor lysate / fluorescence increase of normal lysate (TN ratio) ≥ 2.5; (2) fluorescence increase of tumor lysate in the top 30%.

### Application of probes to fresh specimens

Fresh specimens were divided into 1 mm pieces of tumor and normal tissue, which were placed on µ-Slide 8-Well high Bionert (ibidi GmbH, Gräfelfing, Germany). White-light (measurement wavelength: 600 nm, exposure time: 10 ms) and fluorescence (measurement wavelength: 640 nm, exposure time: 100 ms) images were acquired using the Maestro^®^. 200 µL of probe solution, adjusted to 50 µM, was dropped into each dish. In the case of 2MeSiR, a concentration of 5 µM was used. Fluorescence images were acquired after 1, 3, 5, 10, 20, and 30 min, and the average signal of the ROI (region of interest) was calculated in the 640 nm image. The difference between the values at 3, 5, 10, 20, and 30 min and the value at 1 min was taken as the fluorescence increase, and the values for normal and tumor tissues were obtained. Filter settings were 570/40 nm for excitation and 610 nm for emission. Wavelengths from 600 to 850 nm were measured at intervals of 10 nm. All subsequent measurements were performed with the same settings.

### Comparison of QA-2MeSiR and QA-2OMeSiR

QA-2OMeSiR was obtained in powder form as the trifluoroacetate salt from Goryokayaku Co., Tokyo, Japan. 2OMeSiR was obtained from our university’s stock in powder form^7^. Each probe was dissolved in DMSO and stocked as a 10 mM solution, which was stored in a freezer at −30 °C and used as required. QA-2MeSiR and QA-2OMeSiR were used at the concentration of 50 µM, while 2MeSiR and 2OMeSiR were used at the concentration of 5 µM. Images were obtained with Maestro^®^ and with a digital camera (iPhone 6^®^).

### Observation of borderline lesions using QA-2OMeSiR

In the case of resected specimens that contained relatively large tumors, the transition zones between normal and tumor tissues were sectioned in the pathology department. The fresh samples were placed on a µ-Dish 35 mm, low (ibidi GmbH, Gräfelfing, Germany) and 500 µL of 50 µM QA-2OMeSiR was dropped onto the entire sample.

### Identification of target enzyme candidates by diced electrophoresis gel (DEG) assay and peptide mass fingerprinting (PMF)

DEG assay is a technique developed by Komatsu et al., in which enzyme extracts such as cell lysates are fractionated by nondenaturing two-dimensional electrophoresis. The gels are diced and the pieces are dispensed into multiwell plates, in which enzyme assays using fluorescent probes are performed^15^ (Supplemental Fig. 12). Fluorescence intensity changes were measured by EnVision^®^ immediately and after 12 h. Proteins in gel pieces showing a large fluorescence increase were analyzed by the PMF method at Intégrale Co., Ltd., Tokyo, Japan.

### Reaction of lung cancer lysates with QA-2OMeSiR in the presence of enzyme inhibitors

The following inhibitors were used: sitagliptin (IC50 9.96 nM; DPP4 inhibitor); puromycin (IC50 0.6 µM; PSA inhibitor); AA74-1 (IC50 5 nM; AARE inhibitor); E-64 (IC50 9 nM; bleomycin hydrolase inhibitor); calpain inhibitor II (IC50 120 nM; calpain 1 inhibitor). Final concentrations of inhibitors were 0 M, 1 nM, 10 nM, 100 nM, 1 µM, 10 µM, 100 µM, and 1 mM. The final concentration of QA-2OMeSiR solution was adjusted to 1 µM. 15 µL of the probe and inhibitor were added to wells of a 384-well black plate (Corning, 4511^®^), and the fluorescence intensity was measured by Envision^®^. Then 5 µL of 0.2 mg/ml lysate was added to each well, and the fluorescence was measured every 10 min up to 60 min.

### Reaction of purified enzymes with QA-2OMeSiR

Purified DPP4 (240 ng, MW = 105 kDa) or PSA (228 ng, MW = 100 kDa) was added to QA-2OMeSiR probe solution diluted to 1 µM. Human recombinant DPP4 (D4943 Sigma-Aldrich) and human PSA (6410-ZN-010 R&D Systems) were used. The DPP4 and PSA concentrations were adjusted with PBS ( +) to 0.114 µM (the amounts of DPP4 and PSA were 240 ng and 228 ng, respectively), and the fluorescence increase was monitored with Envision^®^ The total volume of QA-2OMeSiR was 20 µL per well at concentrations of 1, 5, 10, 25, 50, 100, and 200 µM.

### LC–MS analysis

LC–MS analysis was performed using a UPLC-MS system (Waters, Milford, MA). To a PBS solution of 30 mM QA-2OMeSiR was added 274 nM DPP4 (recombinant dipeptidyl peptidase IV human, Catalog number: D4943-1VL, Lot: SLCJ0390, Sigma-Aldrich) or 2 nM PSA (recombinant NPEPPS, Catalog number: 6410-ZN, Lot: SHF0418101, R&D Systems), and the solutions were incubated at 37 °C for the indicated time. The enzyme reaction was quenched by two-fold dilution with MeOH. Aliquots of 10 mL of quenched samples were subjected to UPLC-MS, and the mass and absorbance spectra at 550 nm were analyzed. Gradient elution, A/B = 95/5 to 5/95 (0–3.5 min), 5/95 to 95/5 (4.0–4.1 min), 95/5 (4.1–5.0 min) (eluent A: H_2_O containing 0.1% formic acid, eluent B: acetonitrile containing 0.1% formic acid). For QA-2OMeSiR, *m*/*z* = 558.25; for A-2OMeSiR, *m*/*z* = 430.19; for 2OMeSiR, *m*/*z* = 359.16.

### Imaging of siRNA-transfected lung cancer cell lines with QA-2OMeSiR

To confirm the reactivity of QA-2OMeSiR with DPP4 and PSA, siRNA-transfected lung cancer cells were imaged with QA-2OMeSiR. Lung cancer cell lines (A549, H441, H226) (4 × 10^4^ cells) were seeded in an 8-well chamber (µ-Slide 8 well high Bionert^®^ Ibidi, GmbH, Gräfelfing, Germany) on the day before siRNA transfection. Three DPP4 siRNAs, three PSA siRNAs and a control siRNA was transfected using Opti-MEM and Lipofectamine RNAiMAX transfection reagent (#13778030, Invitrogen). For siRNA sequence information, transfection procedure and reagent preparation, see Supplemental Tables 9 and 10. Cells were incubated in a CO_2_ incubator for 2 days, then QA-2OMeSiR (1 µM) was added and the fluorescence change up to 30 min was observed with an SP8^®^ (Leica Inc., Wetzlar, Germany). The settings were 594 nm for excitation and 600–650 nm for emission.

### Immunohistochemical staining of DPP4 and PSA

Paraffin-embedded sections were washed with water, deparaffinized, and microwaved at 98 °C for 20 min to activate the antigen. The sections were then treated with 3% hydrogen peroxide to block endogenous peroxidase activity, followed by endogenous biotin inactivation using an avidin/biotin blocking kit. The primary antibody CD26 (H-270) (sc-9153 Santa Cruz Biotechnology) was used at a dilution of 1/500 and PSA (sc-390184: mouse monoclonal) was used at a dilution of 1/50. The reaction was carried out for 2 h at room temperature, and counter-stained with hematoxylin.

### Expression of DPP4 and PSA in lung tissue

Frozen specimens of lung tumors and normal tissues were shredded with scissors and RNA was extracted as previously described.Primer sequences for hDPP4 and hPSA, as well as hGAPDH and hHPRT1 (internal standards), are shown in Supplemental Table 11. Other details are shown in Supplemental Table 12. The samples were centrifuged and each sample was measured by triplicate using a LightCycler^®^480 System II (F. Hoffmann-La Roche Ltd, Basel, Switzerland). The mRNA expression levels of DPP4 and PSA were expressed relative to internal standards and compared between tumor and normal tissues.

### Statistical analysis and calculation

ROC curves were drawn using the statistical analysis software R 3.3.2 (R Foundation for Statistical Computing, Vienna, Austria). Comparison between two groups was done with the t-test, and p < 0.05 was considered significant. KaleidaGraph 4.5.3 (HULINKS, Tokyo, Japan) was used to calculate the Michaelis–Menten equation.

### Ethics declarations

This study was approved by the Institutional Ethics Committee of the University of Tokyo ("Ethical Application 3900: Research on the Usefulness of Cancer-Specific Fluorescent Probes and the Development of New Probes in Lung Cancer"). All experiments were performed in accordance with guidelines and regulations approved by the Research Ethics Committee of the University of Tokyo. Informed consent was obtained from all patients. Lung cancer patients examined or treated at the University of Tokyo Hospital in Tokyo, Japan, were prospectively included in this study.

## Supplementary Information

Supplementary Information.

## Data Availability

The authors confirm that the data supporting the findings of this study are available within the article and its supplementary information.
