# Supplementary material for: Rapid imaging of lung cancer using a red fluorescent probe to detect dipeptidyl peptidase 4 and puromycin-sensitive aminopeptidase activities
Source: Sci Rep. 2022 Jun 1;12:9100. doi: 10.1038/s41598-022-12665-9 (PMC9160295; doi:10.1038/s41598-022-12665-9)
Supplement: Supplementary file 1 — Supplementary Information. [file 41598_2022_12665_MOESM1_ESM.pdf]

Supplementary information for Rapid imaging of lung cancer using a red fluorescent probe to detect dipeptidyl peptidase 4 and puromycin-sensitive aminopeptidase activities

Authors: Shun Kawashima, Daisuke Yoshida, Takafusa Yoshioka, Akira Ogasawara, Kyohhei Fujita, Masahiro Yanagiya, Masaaki Nagano, Chihiro Konoeda, Haruaki Hino, Kentaro Kitano, Masaaki Sato, Rumi Hino, Ryosuke Kojima, Toru Komatsu, Mako Kamiya, Yasuteru Urano* and Jun Nakajima*
*To whom correspondence should be addressed. E-mail: uranokun@m.u-tokyo.ac.jp / nakajima-tho@h.u-tokyo.ac.jp

This file includes:

Supplemental Figure 1. Chemical structures of gGlu-HMRG, HMRG, (peptide-)2MeSiR and (peptide-)2OMeSiR.
Supplemental Figure 2. Time course of fluorescence intensity of selected probes (QA-2MeSiR, AY-2MeSiR and GL-2MeSiR) in adenocarcinoma lysate screening.
Supplemental Figure 3. Structural formulae and cleavage reaction of QA-2MeSiR and QA-2OMeSiR.
Supplemental Figure 4. Images showing DEG assay results.
Supplemental Figure 5. Fluorescence of adenocarcinoma lysate and QA-2OMeSiR in the presence of various concentrations of sitagliptin (DPP4 inhibitor).
Supplemental Figure 6. Fluorescence of adenocarcinoma lysate and QA-2OMeSiR in the presence of various concentrations of puromycin (PSA inhibitor).
Supplemental Figure 7. Fluorescence of adenocarcinoma lysate and QA-2OMeSiR in the presence of various concentrations of AA74-1 (AARE inhibitor).
Supplemental Figure 8. Fluorescence of adenocarcinoma lysate and QA-2OMeSiR in the presence of various concentrations of E-64 (bleomycin hydrolase inhibitor).
Supplemental Figure 9. Fluorescence of adenocarcinoma lysate and QA-2OMeSiR in the presence of various concentrations of calpain inhibitor II (calpain 1 inhibitor).
Supplemental Figure 10. LC-MS analysis of the reactions of QA-2OMeSiR with recombinant DPP4 and PSA.
Supplemental Figure 11. Schematic illustration of the conversion of QA-2OMeSiR to 2OMeSiR in lung cancer cells
Supplemental Figure 12. Work-flow of diced electrophoresis gel (DEG) assay.

Supplemental Table 1. Amino acid sequences of the 400 fluorescent probes
Supplemental Table 2. One-letter abbreviations of amino acids
Supplemental Table 3. The 62 probes meeting the lysate screening criteria
Supplemental Table 4. AUC, specificity and sensitivity of nine selected probes applied to fresh samples.
Supplemental Table 5. Results of applying QA-2MeSiR to 27 fresh lung cancer specimens
Supplemental Table 6. Results of peptide mass fingerprinting (PMF) analyses ①
Supplemental Table 7. Results of PMF analyses ②
Supplemental Table 8. Results of PMF analyses ③
Supplemental Table 9. siRNA sequences
Supplemental Table 10. Preparation of siRNA reagents
Supplemental Table 11. Sequences of primers used for quantitative PCR
Supplemental Table 12. Reagents for quantitative PCR

Supplemental Figures.

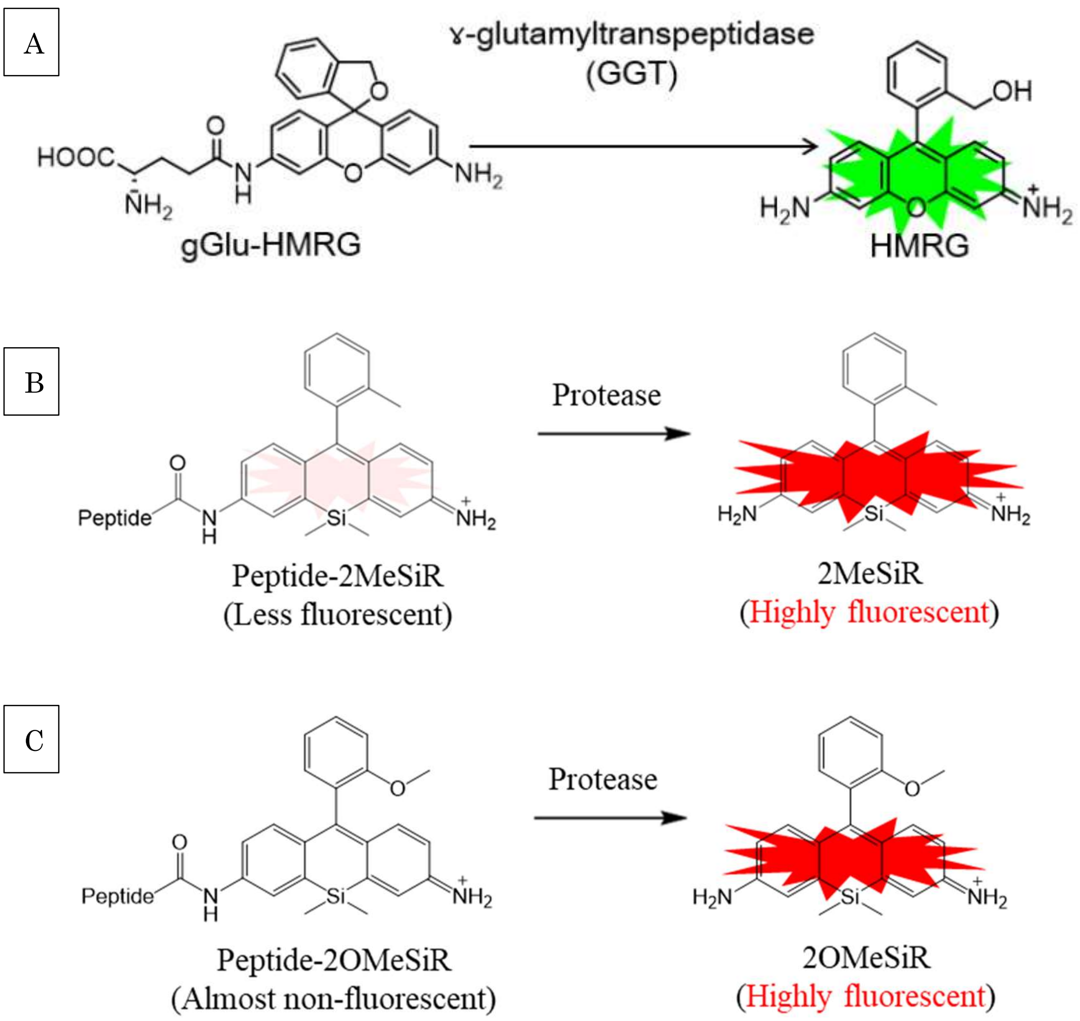

Supplemental Figure 1. Chemical structures of gGlu-HMRG, HMRG, (Peptide-)2MeSiR and (Peptide-)2OMeSiR. **A.** gGlu-HMRG does not fluoresce, but is hydrolyzed by GGT to HMRG, which emits strong fluorescence. **B.** 2MeSiR is a red fluorescent structure with an absorption maximum at 593 nm and a fluorescence maximum at 613 nm. When a peptide chain is attached to one of the amino groups of the xanthene ring, the absorption wavelength shifts to the shorter wavelength side. Therefore, the probe is hardly excited at around 590 nm and does not emit fluorescence, but after the peptide chain is cleaved, the fluorophore can be excited efficiently and emits strong fluorescence. **C.** 2-Methoxy silicon rhodamine (2OMeSiR) was synthesized to increase the fluorescence activation ratio; the fluorescence of the intact probe is strongly quenched by photo-induced electron transfer, but strong fluorescence is emitted after the peptide bond is cleaved.

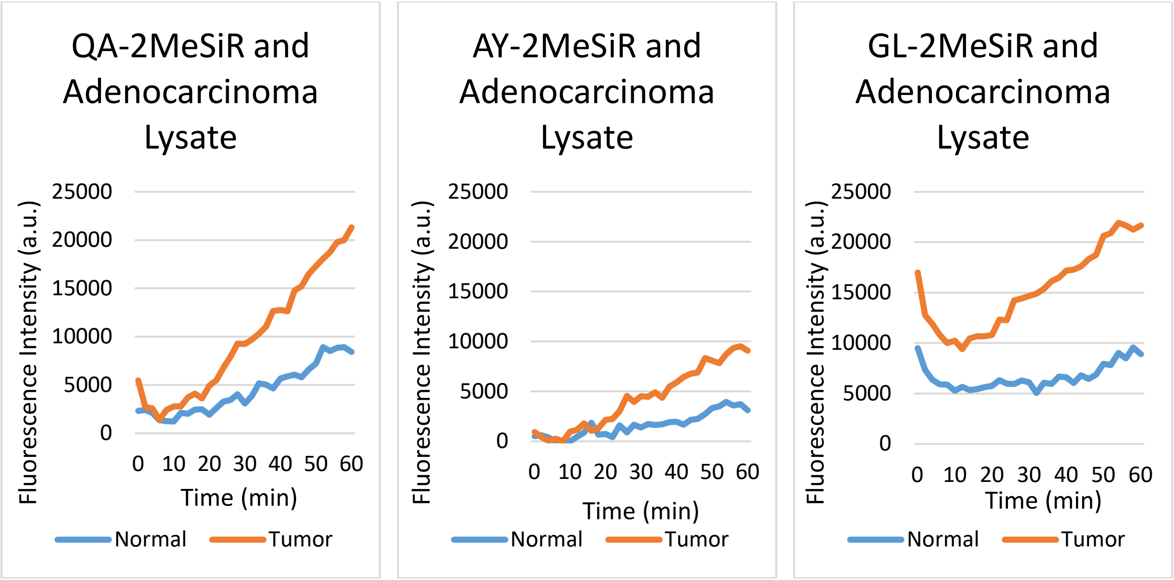

Supplemental Figure 2. Time course of fluorescence intensity of selected probes (QA-2MeSiR, AY-2MeSiR and GL-2MeSiR) in adenocarcinoma lysate screening.

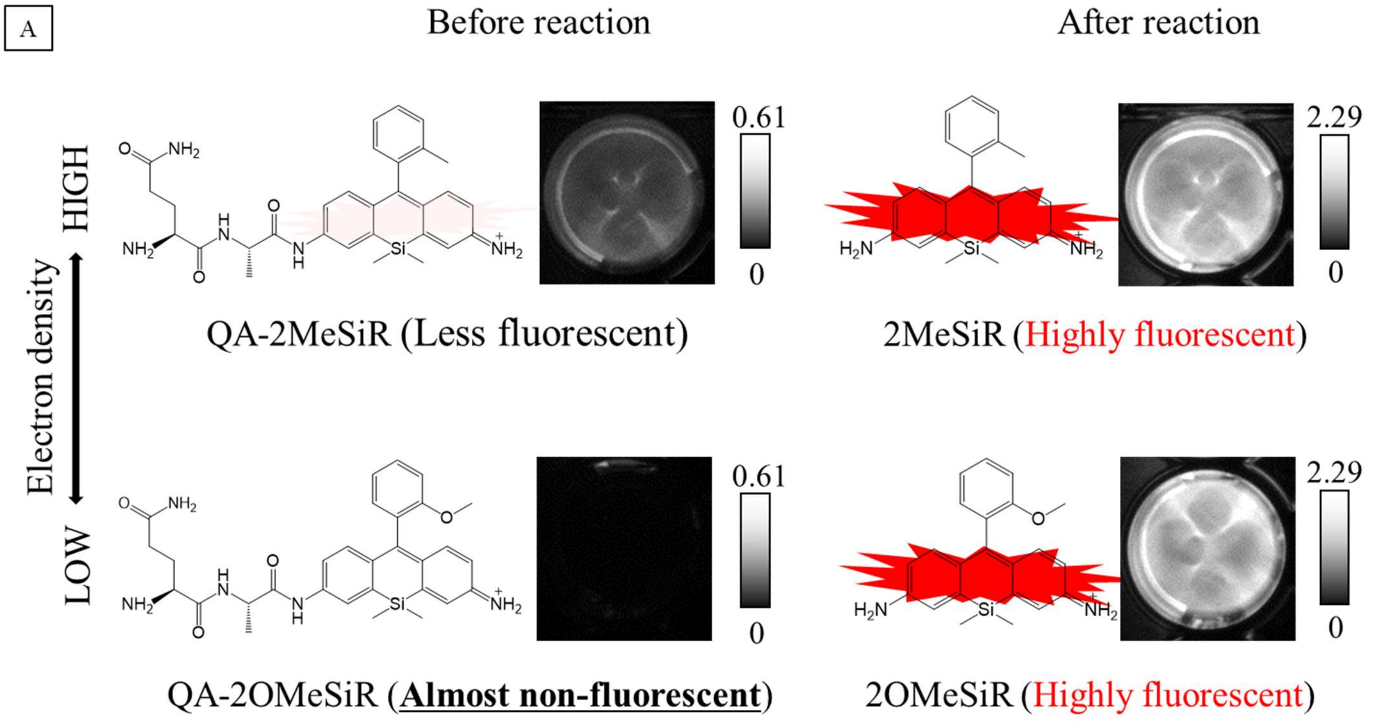

B

QA-2MeSiR	2MeSiR
QA-2OMeSiR	2OMeSiR

C

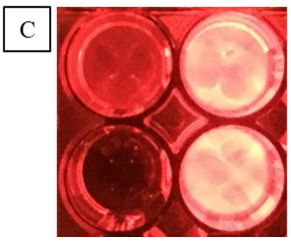

D

Fluorescence Intensity		Ratio
0.124	1.28	x 10.3
0.025	1.32	x 52.8

Supplemental Figure 3. Structural formulae and cleavage reaction of QA-2MeSiR and QA-2OMeSiR, fluorescence photograph at 640 nm and digital camera image. **A.** The concentrations of QA-2MeSiR and QA-2OMeSiR were adjusted to 50 µM, and those of 2MeSiR and 2OMeSiR were adjusted to 5 µM. 400 µL of each probe solution was dropped into wells of a 48-well plate and the plate was photographed with Maestro®. The values on the right show the fluorescence intensity range (a.u.) at 640 nm at the time of imaging. **B.** Names of the fluorescent probes used in the corresponding wells of part C. **C.** Photo taken with a digital camera (iPhone6®) with no correction except for a filter. **D.** Fluorescence intensity of each probe. The fluorescence intensity of 2OMeSiR was 52.8 times higher than that of QA-2OMeSiR, while that of 2MeSiR was 10.3 times higher than that of QA-2MeSiR (Supplemental Figure 3D).

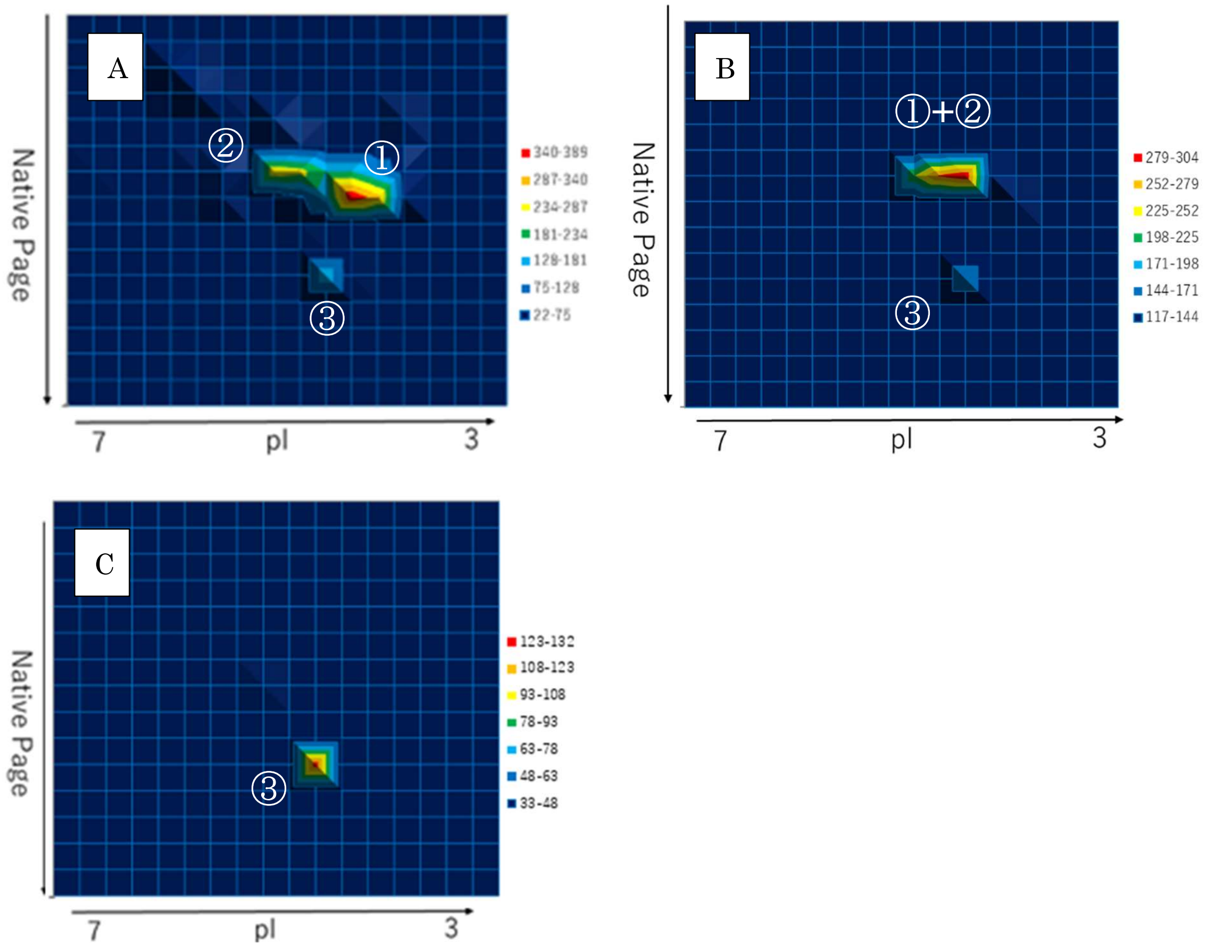

Supplemental Figure 4. The results of DEG assay. **A.** The reaction of QA-2OMeSiR and adenocarcinoma lysate (#5) (0.68 mg/ml). Two peaks (① and ②) on the upper side, and a small peak (③) on the lower side were recognized. **B.** The reaction of QA-2OMeSiR and another adenocarcinoma lysate (#10) (1.00 mg/ml). Similar peaks were observed in both adenocarcinoma lysates. The upper peaks were fused (①+②), and the small lower peaks were similar (③).

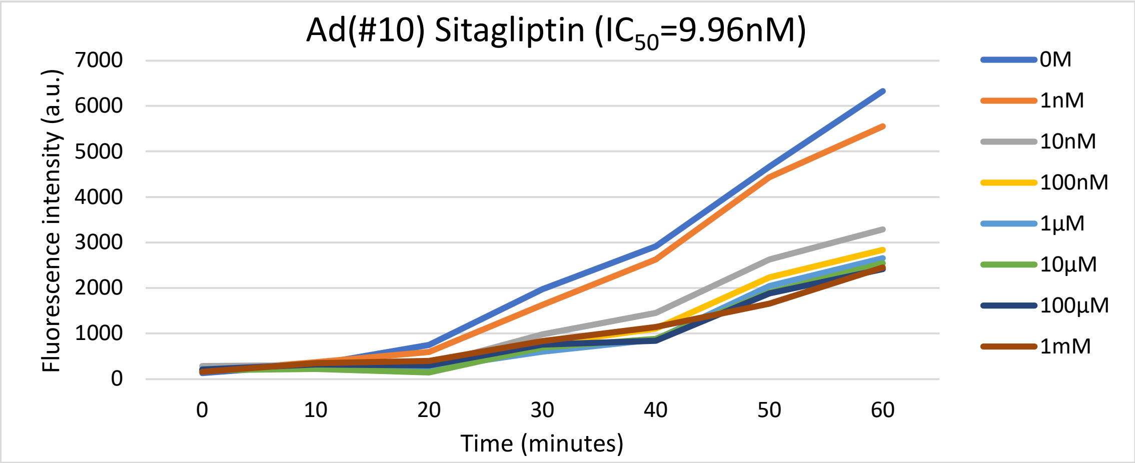

Supplemental Figure 5. Fluorescence of adenocarcinoma lysate and QA-2OMeSiR in the presence of various concentrations of sitagliptin (DPP4 inhibitor). Sitagliptin above 10 nM (above the IC_{50}) suppressed the fluorescence increase.

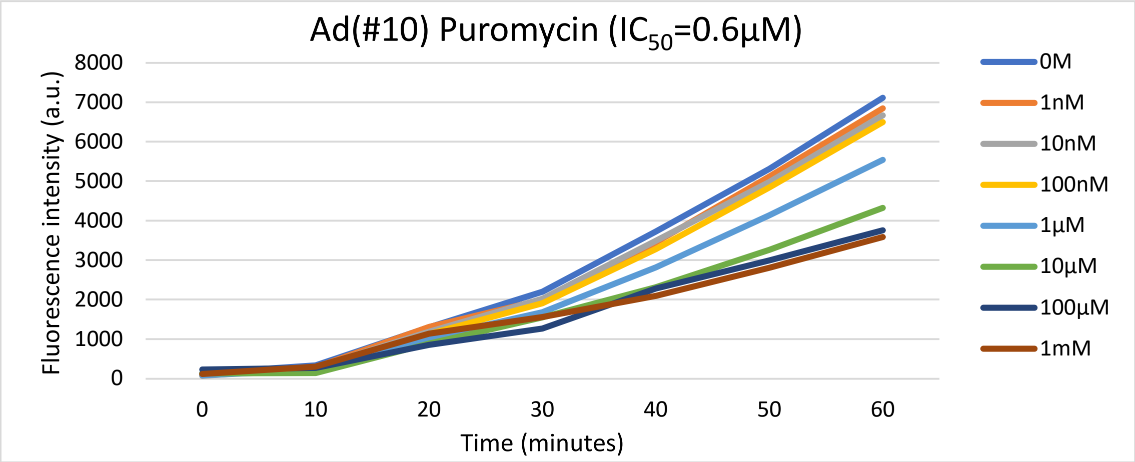

Supplemental Figure 6. Fluorescence of adenocarcinoma lysate and QA-2OMeSiR in the presence of various concentrations of puromycin (PSA inhibitor). Puromycin above 1µM (above the IC_{50}) suppressed the fluorescence increase.

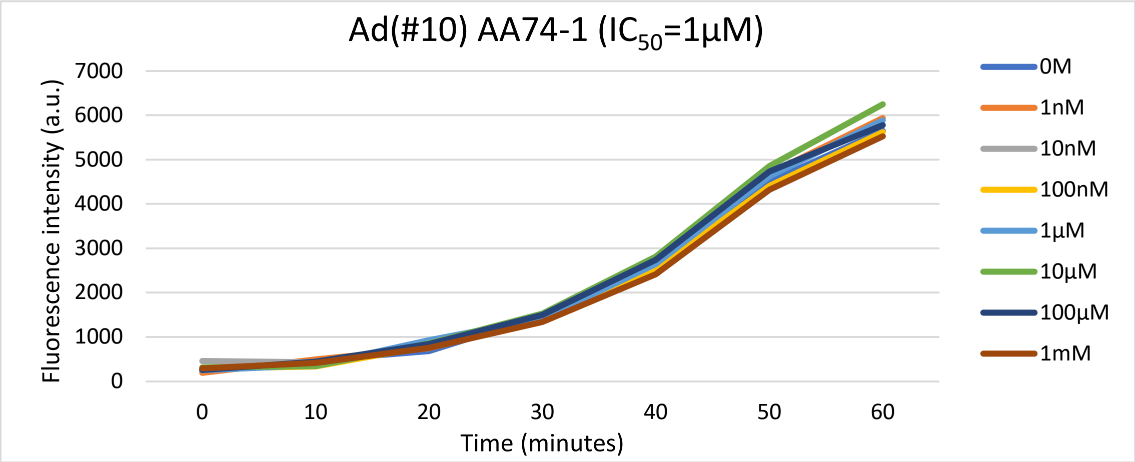

Supplemental Figure 7. Fluorescence of adenocarcinoma lysate and QA-2OMeSiR in the presence of various concentrations of AA74-1 (AARE inhibitor). AA74-1 was not inhibitory.

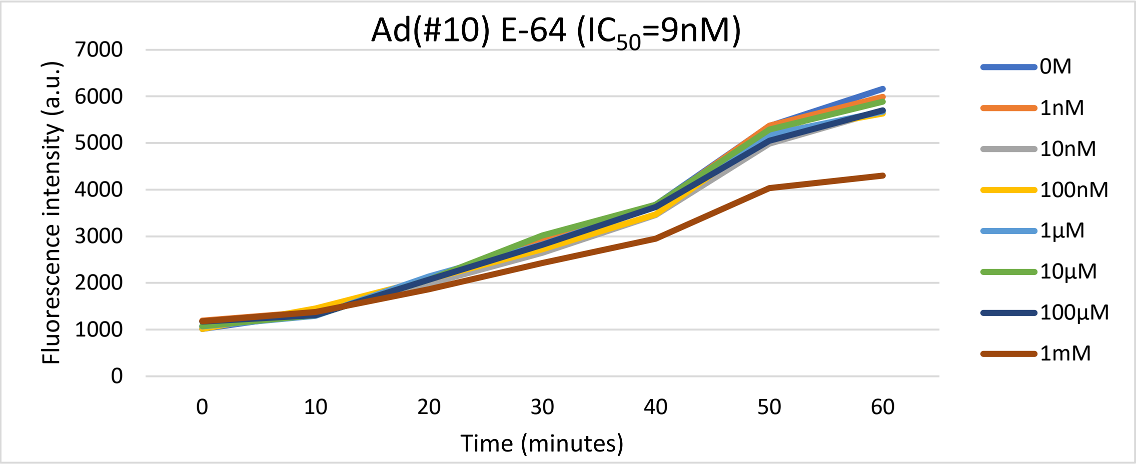

Supplemental Figure 8. Fluorescence of adenocarcinoma lysate and QA-2OMeSiR in the presence of various concentrations of E-64 (bleomycin hydrolase inhibitor). E-64 was not inhibitory.

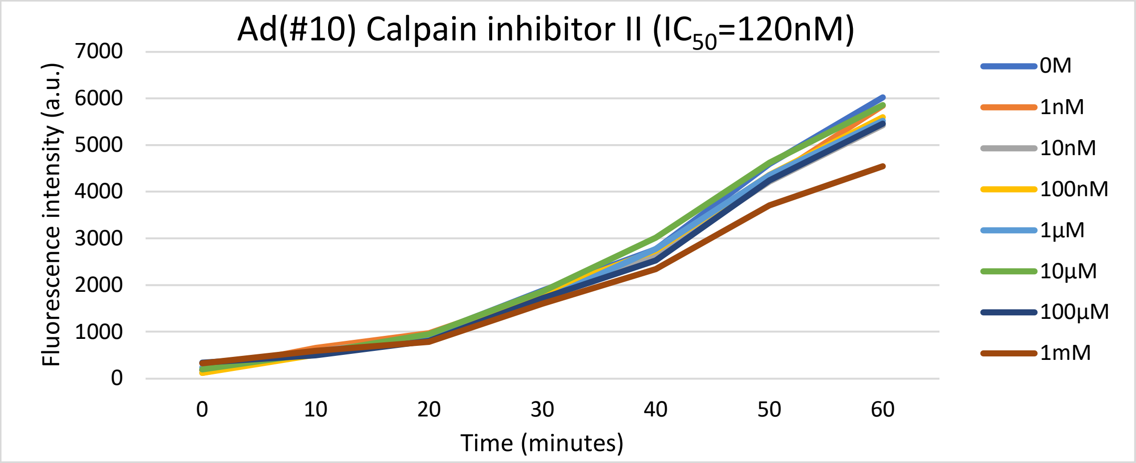

Supplemental Figure 9. Fluorescence of adenocarcinoma lysate and QA-2OMeSiR in the presence of various concentrations of calpain inhibitor II (calpain 1 inhibitor). Calpain inhibitor II was not inhibitory.

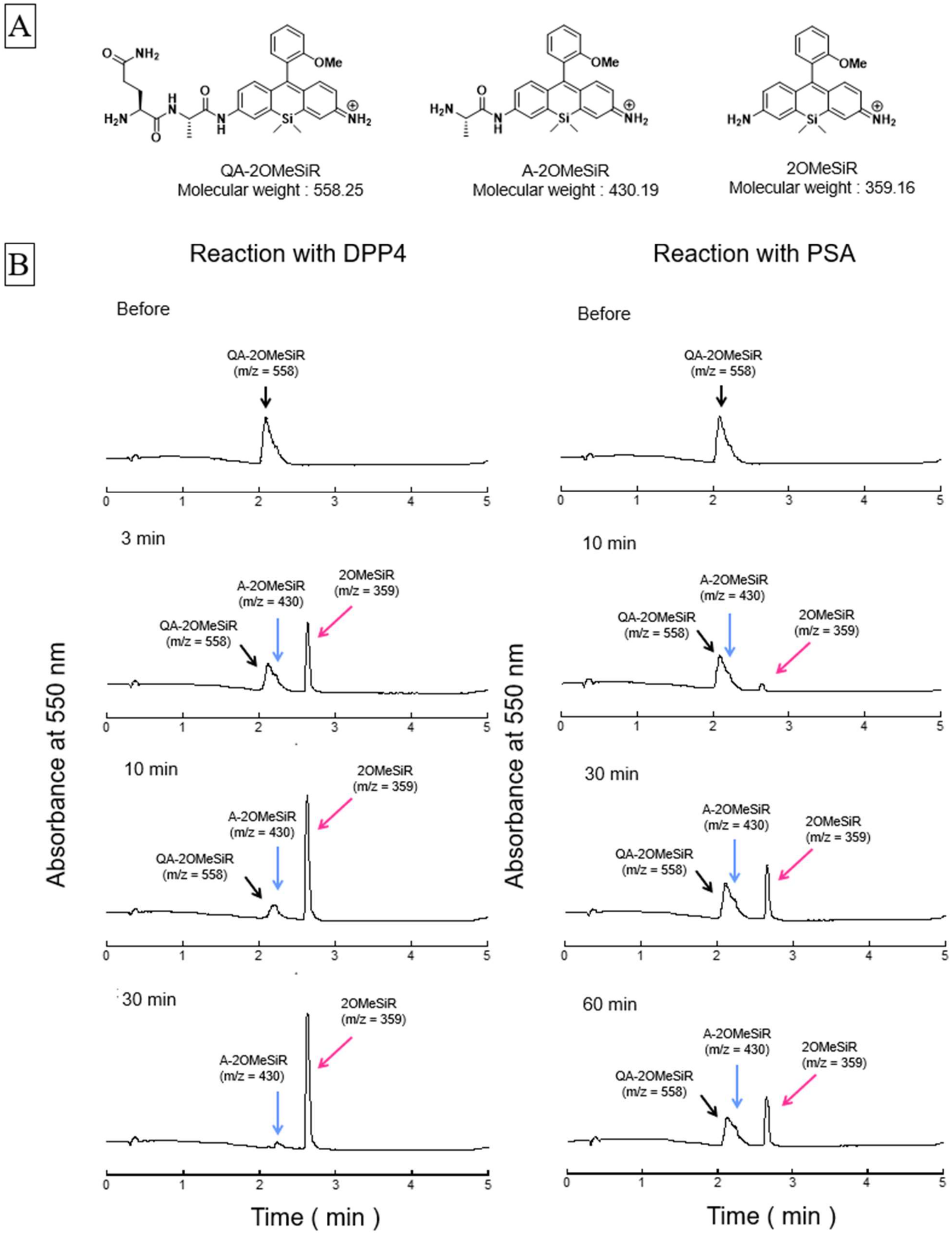

Supplemental Figure 10. Analysis of the reactions of QA-2OMeSiR with recombinant DPP4 and PSA. (**A**) Chemical structure and molecular weight of QA-2OMeSiR, A-2OMeSiR and 2OMeSiR. (**B**) Results of UPLC-MS analysis of the hydrolysis products of QA-2OMeSiR generated by DPP4 and PSA. The enzyme reactions both proceed rapidly, affording highly fluorescent 2OMeSiR.

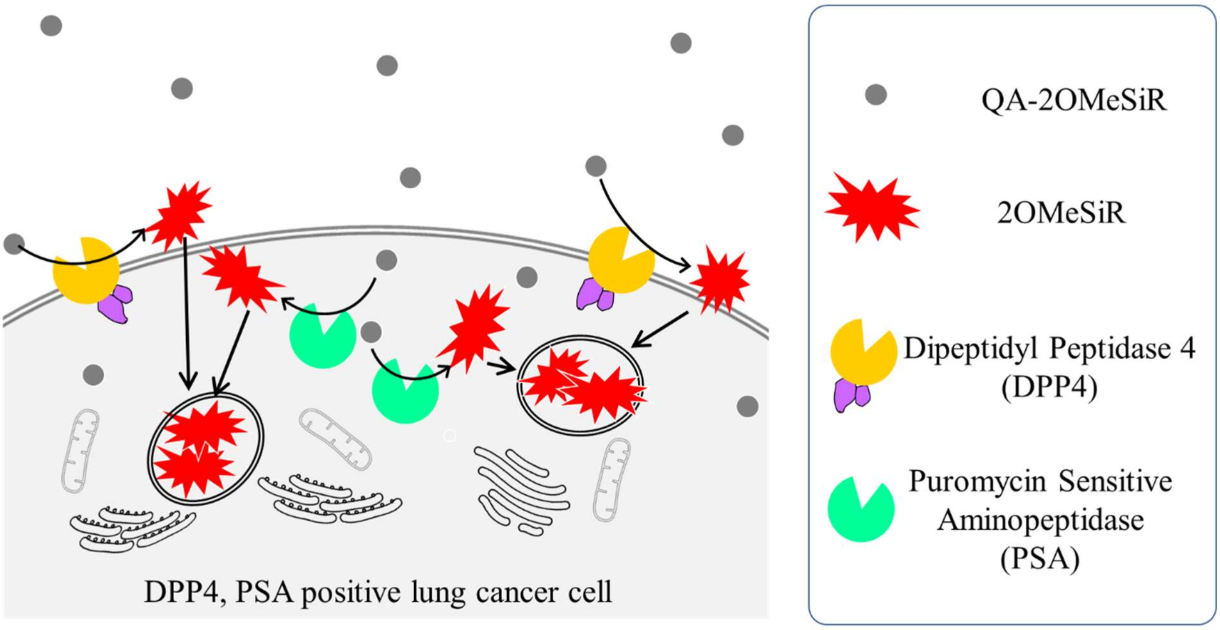

Supplemental Figure 11. Schematic illustration of the conversion of QA-2OMeSiR to 2OMeSiR in lung cancer cells. Non-fluorescent QA-2OMeSiR is converted to highly fluorescent 2OMeSiR by the enzymatic activity of DPP4, a membrane protein expressed in lung cancer cells, and/or PSA, a cytoplasmic protein. 2OMeSiR is then taken up by lysosomes.

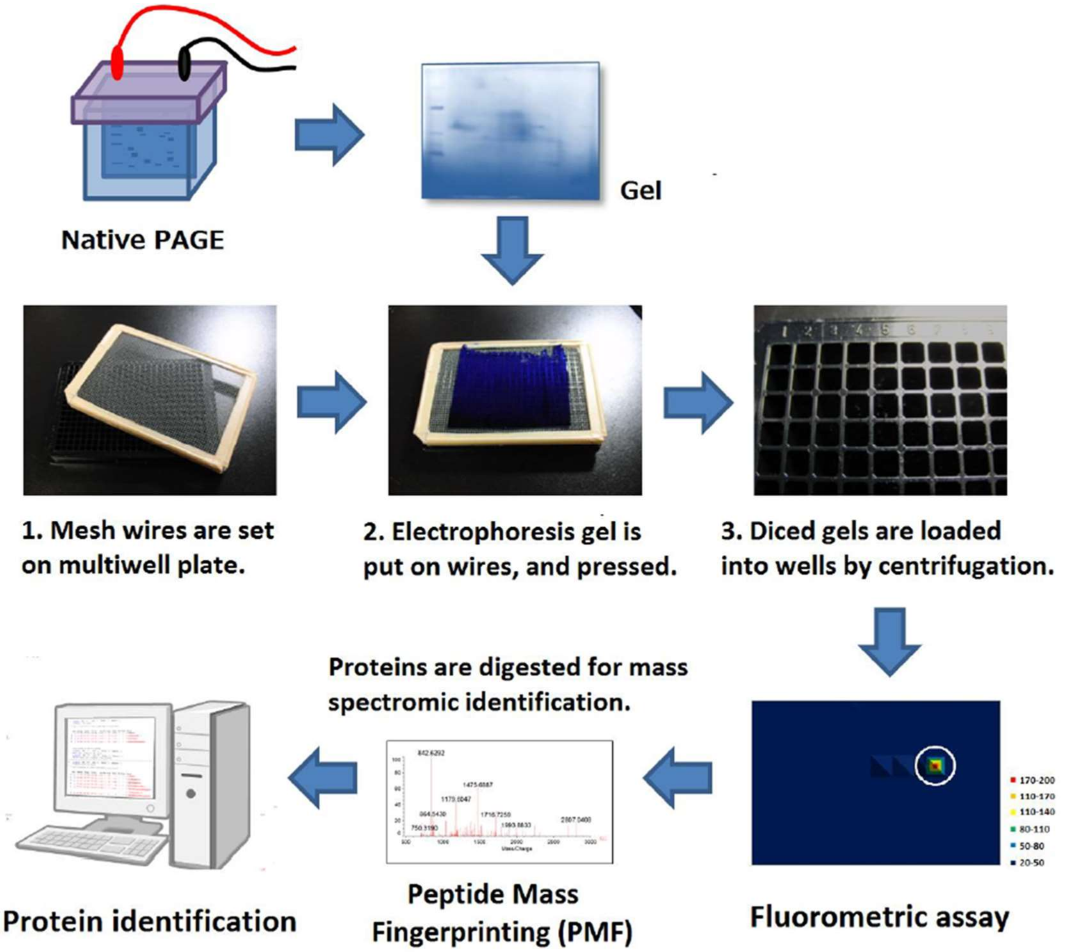

Supplemental Figure 12. Work-flow of DEG assay (cited from [15]). Two-dimensional electrophoresis of the tumor lysate based on molecular weight and isoelectric point is performed, and the gel is diced. The pieces are dropped into wells of a multiwell plate for fluorescence assay with fluorescent probes. Proteins in the wells with high fluorescence are target enzyme candidates, and are identified by mass fingerprinting.

Supplemental Tables

Supplemental Table 1 Amino acid sequences of the 400 fluorescent probes. The assigned number of the probe in the library and the amino acid sequence of the side chain are shown.

P2 \ P1	G	E	K	Y	L	P	I	A	V	W
G	1 G G	11 G E	21 G K	31 G Y	41 G L	51 G P	61 G I	71 G A	81 G V	91 G W
E	2 E G	12 E E	22 E K	32 E Y	42 E L	52 E P	62 E I	72 E A	82 E V	92 E W
K	3 K G	13 K E	23 K K	33 K Y	43 K L	53 K P	63 K I	73 K A	83 K V	93 K W
Y	4 Y G	14 Y E	24 Y K	34 Y Y	44 Y L	54 Y P	64 Y I	74 Y A	84 Y V	94 Y W
L	5 L G	15 L E	25 L K	35 L Y	45 L L	55 L P	65 L I	75 L A	85 L V	95 L W
P	6 P G	16 P E	26 P K	36 P Y	46 P L	56 P P	66 P I	76 P A	86 P V	96 P W
I	7 I G	17 I E	27 I K	37 I Y	47 I L	57 I P	67 I I	77 I A	87 I V	97 I W
A	8 A G	18 A E	28 A K	38 A Y	48 A L	58 A P	68 A I	78 A A	88 A V	98 A W
V	9 V G	19 V E	29 V K	39 V Y	49 V L	59 V P	69 V I	79 V A	89 V V	99 V W
W	10 W G	20 W E	30 W K	40 W Y	50 W L	60 W P	70 W I	80 W A	90 W V	100 W W
T	101 T G	111 T E	121 T K	131 T Y	141 T L	151 T P	161 T I	171 T A	181 T V	191 T W
H	102 H G	112 H E	122 H K	132 H Y	142 H L	152 H P	162 H I	172 H A	182 H V	192 H W
S	103 S G	113 S E	123 S K	133 S Y	143 S L	153 S P	163 S I	173 S A	183 S V	193 S W
F	104 F G	114 F E	124 F K	134 F Y	144 F L	154 F P	164 F I	174 F A	184 F V	194 F W
C	105 C G	115 C E	125 C K	135 C Y	145 C L	155 C P	165 C I	175 C A	185 C V	195 C W
M	106 M G	116 M E	126 M K	136 M Y	146 M L	156 M P	166 M I	176 M A	186 M V	196 M W
N	107 N G	117 N E	127 N K	137 N Y	147 N L	157 N P	167 N I	177 N A	187 N V	197 N W
Q	108 Q G	118 Q E	128 Q K	138 Q Y	148 Q L	158 Q P	168 Q I	178 Q A	188 Q V	198 Q W
D	109 D G	119 D E	129 D K	139 D Y	149 D L	159 D P	169 D I	179 D A	189 D V	199 D W
R	110 R G	120 R E	130 R K	140 R Y	150 R L	160 R P	170 R I	180 R A	190 R V	200 R W

P2 \ P1	T	H	S	F	C	M	N	Q	D	R
G	201 G T	211 G H	221 G S	231 G F	241 G C	251 G M	261 G N	271 G Q	281 G D	291 G R
E	202 E T	212 E H	222 E S	232 E F	242 E C	252 E M	262 E N	272 E Q	282 E D	292 E R
K	203 K T	213 K H	223 K S	233 K F	243 K C	253 K M	263 K N	273 K Q	283 K D	293 K R
Y	204 Y T	214 Y H	224 Y S	234 Y F	244 Y C	254 Y M	264 Y N	274 Y Q	284 Y D	294 Y R
L	205 L T	215 L H	225 L S	235 L F	245 L C	255 L M	265 L N	275 L Q	285 L D	295 L R
P	206 P T	216 P H	226 P S	236 P F	246 P C	256 P M	266 P N	276 P Q	286 P D	296 P R
I	207 I T	217 I H	227 I S	237 I F	247 I C	257 I M	267 I N	277 I Q	287 I D	297 I R
A	208 A T	218 A H	228 A S	238 A F	248 A C	258 A M	268 A N	278 A Q	288 A D	298 A R
V	209 V T	219 V H	229 V S	239 V F	249 V C	259 V M	269 V N	279 V Q	289 V D	299 V R
W	210 W T	220 W H	230 W S	240 W F	250 W C	260 W M	270 W N	280 W Q	290 W D	300 W R
T	301 T T	311 T H	321 T S	331 T F	341 T C	351 T M	361 T N	371 T Q	381 T D	391 T R
H	302 H T	312 H H	322 H S	332 H F	342 H C	352 H M	362 H N	372 H Q	382 H D	392 H R
S	303 S T	313 S H	323 S S	333 S F	343 S C	353 S M	363 S N	373 S Q	383 S D	393 S R
F	304 F T	314 F H	324 F S	334 F F	344 F C	354 F M	364 F N	374 F Q	384 F D	394 F R
C	305 C T	315 C H	325 C S	335 C F	345 C C	355 C M	365 C N	375 C Q	385 C D	395 C R
M	306 M T	316 M H	326 M S	336 M F	346 M C	356 M M	366 M N	376 M Q	386 M D	396 M R
N	307 N T	317 N H	327 N S	337 N F	347 N C	357 N M	367 N N	377 N Q	387 N D	397 N R
Q	308 Q T	318 Q H	328 Q S	338 Q F	348 Q C	358 Q M	368 Q N	378 Q Q	388 Q D	398 Q R
D	309 D T	319 D H	329 D S	339 D F	349 D C	359 D M	369 D N	379 D Q	389 D D	399 D R
R	310 R T	320 R H	330 R S	340 R F	350 R C	360 R M	370 R N	380 R Q	390 R D	400 R R

Supplemental Table 2 One-letter abbreviations of amino acids

Code	Name	Code	Name	Code	Name	Code	Name
A	Alanine	G	Glycine	M	Methionine	S	Serine
C	Cysteine	H	Histidine	N	Asparagine	T	Threonine
D	Aspartic Acid	I	Isoleucine	P	Proline	V	Valine
E	Glutamic Acid	K	Lysine	Q	Glutamine	W	Tryptophan
F	Phenylalanine	L	Leucine	R	Arginine	Y	Tyrosine

Supplemental Table 3 The 62 probes meeting the lysate screening criteria. The number of cases meeting the criteria (increase of fluorescence of tumor lysate/that of normal lysate ≥ 2.5, and increase of fluorescence of tumor lysate in the top 30%) is shown.

Positive Cases (n=11)	No.	P2P1	Positive Cases (n=11)	No.	P2P1	Positive Cases (n=11)	No.	P2P1	Positive Cases (n=11)	No.	P2P1
7	178	QA	5	337	NF	4	51	GP	4	191	TW
6	38	AY	5	254	YM	4	52	EP	4	193	SW
6	41	GL	4	23	KK	4	59	VP	4	236	PF
6	77	IA	4	24	YK	4	156	MP	4	332	HF
6	79	VA	4	25	LK	4	158	QP	4	336	MF
5	31	GY	4	27	IK	4	63	KI	4	251	GM
5	45	LL	4	29	VK	4	65	LI	4	257	IM
5	54	YP	4	34	YY	4	68	AI	4	259	VM
5	55	LP	4	133	SY	4	164	FI	4	357	NM
5	58	AP	4	134	FY	4	166	MI	4	274	YQ
5	153	SP	4	136	MY	4	170	RI	4	300	WR
5	154	FP	4	43	KL	4	73	KA	4	394	FR
5	160	RP	4	44	YL	4	74	YA	4	398	QR
5	196	MW	4	50	WL	4	75	LA	4	400	RR
5	331	TF	4	143	SL	4	80	WA			
5	333	SF	4	146	ML	4	174	FA			

Supplemental Table 4. AUC, specificity and sensitivity of nine selected probes and pure 2MeSiR applied to fresh samples. QA-2MeSiR showed the best result in all cases.

		Primary lung cancer							Non-small cell lung cancer						
No.	P2P1	n(N)	n(T)	AUC	95%CI	cut off	Sp.	Se.	n(N)	n(T)	AUC	95%CI	cut off	Sp.	Se.
178	QA	18	18	0.883	0.762-1	0.821	0.778	0.944	17	17	0.882	0.758-1	0.842	0.765	0.824
333	SF	13	13	0.828	0.666-0.991	0.475	0.846	0.769	12	12	0.854	0.697-1	0.475	0.833	0.833
38	AY	17	17	0.827	0.69-0.964	0.723	0.706	0.588	15	15	0.818	0.666-0.969	0.723	0.667	0.6
34	YY	12	12	0.812	0.629-0.996	0.21	0.667	0.917	11	11	0.793	0.594-0.993	0.21	0.636	0.909
331	TF	15	15	0.796	0.628-0.963	0.631	0.933	0.667	13	13	0.822	0.647-0.998	0.631	0.923	0.769
41	GL	17	17	0.782	0.624-0.94	0.787	0.706	0.706	16	16	0.809	0.656-0.961	0.862	0.75	0.688
35	LY	9	9	0.765	0.528-1	0.389	0.778	0.778	8	8	0.766	0.504-1	0.389	0.75	0.875
337	NF	13	13	0.763	0.541-0.979	0.627	0.923	0.615	12	12	0.736	0.528-0.944	0.674	0.917	0.583
133	SY	10	10	0.74	0.5-0.98	0.467	0.7	0.9	7	7	0.714	0.403-1	0.467	0.714	0.857
2MeSiR		19	19	0.476	0.282-0.671	0.272	0.842	0.316	18	18	0.475	0354-0.793	0.272	0.833	0.333

		Adenocarcinoma							Squamous cell carcinoma						
No.	P2P1	n(N)	n(T)	AUC	95%CI	cut off	Sp.	Se.	n(N)	n(T)	AUC	95%CI	cut off	Sp.	Se.
178	QA	13	13	0.864	0.711-1	0.837	0.769	0.923	3	3	1	1-1	0.825	1	1
333	SF	10	10	0.86	0.679-1	0.475	0.9	0.8	1	1	1	NaN-NaN	0.482	1	1
38	AY	13	13	0.84	0.687-0.994	0.616	0.692	0.923	2	2	1	1-1	0.355	1	1
34	YY	8	8	0.828	0.597-1	0.324	0.875	0.625	2	2	1	1-1	0.238	1	1
331	TF	12	12	0.847	0.671-1	0.631	1	0.75	1	1	1	NaN-NaN	0.506	1	1
41	GL	13	13	0.805	0.648-1	0.643	0.615	0.923	3	3	0.889	0.581-1	0.677	0.667	0.667
35	LY	7	7	0.837	0.601-1	0.389	0.857	0.857	0	0	-	-	-	-	-
337	NF	8	8	0.922	0.762-1	0.674	1	0.875	3	3	0.556	0-1	0.153	0.333	1
133	SY	8	8	0.781	0.534-1	0.467	0.75	0.875	0	0	-	-	-	-	-
2MeSiR		12	12	0.542	0.221-0.779	0.319	0.833	0.417	5	5	0.76	0.372-1	0.105	1	0.6

Supplemental Table 5. Results of applying QA-2MeSiR to 27 fresh lung cancer specimens. In all lung cancers, the highest AUC was observed at 10 minutes after probe application. No significant difference in AUC was observed between different tissues.

	Lung cancer (N=27)	NSCLC (N=26)	Adenocarcinoma (N=19)	SCC (N=5)
5min				
AUC	0.960	0.960	0.970	0.960
95%CI	0.915-1	0.915-1	0.92-1	0.849-1
Cutoff (a.u.)	0.072	0.072	0.072	0.082
Sensitivity (%)	92.6	92.3	94.7	80
Specificity (%)	92.6	92.3	94.7	100
Average T/N	2.79	2.81	2.83	2.48
Cases (T/N>2)	20 (74%)	19 (73%)	15 (79%)	3 (60%)
10min				
AUC	0.962	0.959	0.961	0.960
95%CI	0.92-1	0.913-1	0.909-1	0.849-1
Cutoff	0.187	0.187	0.187	0.277
Sensitivity (%)	96.3	96.2	94.7	80
Specificity (%)	85.2	84.6	89.5	100
Average T/N	2.93	2.94	2.89	2.52
Cases (T/N>2)	21 (78%)	20 (77%)	15 (79%)	4 (80%)
20min				
AUC	0.929	0.930	0.922	1
95%CI	0.862-0.995	0.864-0.997	0.839-1	1-1
Cutoff	0.539	0.539	0.473	0.539
Sensitivity (%)	96.3	96.2	94.7	100
Specificity (%)	81.5	80.8	73.7	100
Average T/N	2.90	2.92	2.67	2.79
Cases (T/N>2)	17 (63%)	16 (62%)	12 (63%)	3 (60%)
30min				
AUC	0.916	0.917	0.892	1
95%CI	0.832-1	0.831-1	0.775-1	1-1
Cutoff	0.821	0.823	0.842	0.825
Sensitivity (%)	96.3	96.2	89.5	100
Specificity (%)	81.5	80.8	78.9	100
Average T/N	2.46	2.48	2.26	2.51
Cases (T/N>2)	15 (56%)	15 (58%)	11 (58%)	3 (60%)

Supplemental Table 6. Result of peptide mass fingerprinting (PMF) analyses ① (Upper right hot spot in DEG assay). Enzymes with activity to hydrolyze peptide bonds are highlighted in yellow, and those considered to have potential probe-hydrolyzing activity are highlighted in orange.

①	Identified Enzymes	Protein Identification Probability
1	Ubiquitin-like modifier-activating enzyme 1	100%
2	Acylamino-acid-releasing enzyme	100%
3	Protein disulfide-isomerase	100%
4	Glyceraldehyde-3-phosphate dehydrogenase	100%
5	Creatine kinase B-type	100%
6	Dipeptidyl peptidase 4	100%
7	Lysozyme C	100%
8	Ubiquitin carboxyl-terminal hydrolase 5	99%
9	Trypsin-1	95%
10	Trypsin-3	95%
11	Biliverdin reductase A	95%
12	Inositol-3-phosphate synthase 1	95%
13	Ceruloplasmin	94%
14	Arginase-1	5%

Supplemental Table 7. Result of PMF analyses ② (Upper left hot spot on DEG assay). Enzymes with activity to hydrolyze peptide bonds are highlighted in yellow, and those considered to have potential probe-hydrolyzing activity are highlighted in orange.

②	Identified Enzymes	Protein Identification Probability
1	Retinal dehydrogenase 1	100%
2	Glyceraldehyde-3-phosphate dehydrogenase	100%
3	Cluster of Dihydropyrimidinase-related protein 2	100%
4	Methanethiol oxidase	100%
5	Aldehyde dehydrogenase, mitochondrial	100%
6	Triosephosphate isomerase	100%
7	Cullin-4B	100%
8	L-lactate dehydrogenase A chain	100%
9	Succinyl-CoA:3-ketoacid coenzyme A transferase 1, mitochondrial	100%
10	Glutamate dehydrogenase 1, mitochondrial	100%
11	Protein disulfide-isomerase A3	100%
12	Delta-aminolevulinic acid dehydratase	100%
13	4-trimethylaminobutyraldehyde dehydrogenase	100%
14	Glycogen phosphorylase, brain form	100%
15	L-lactate dehydrogenase B chain	100%
16	Calpain-1 catalytic subunit	99%
17	Protein-glutamine gamma-glutamyltransferase K	100%
18	Lysozyme C	100%
19	Tissue alpha-L-fucosidase	100%
20	Tryptase beta-2	100%
21	Caspase-14	100%
22	Protein-glutamine gamma-glutamyltransferase E	100%
23	Trypsin-3	95%
24	UDP-glucose:glycoprotein glucosyltransferase 1	91%
25	Cathepsin D	84%
26	Transaldolase	84%
27	Trypsin-1	65%
28	Bleomycin hydrolase	47%
29	Glyoxylate reductase/hydroxypyruvate reductase	42%
30	Dipeptidyl peptidase 4	29%
31	Coagulation factor XIII A chain	19%
32	Cytosolic non-specific dipeptidase	10%

Supplemental Table 8. Result of PMF analyses ③ (Lower hot spot on DEG assay). The only enzyme with potential probe-hydrolyzing activity is highlighted in orange.

③	Identified Enzymes	Protein Identification Probability
1	Puromycin-sensitive aminopeptidase	100%
2	Calpain-2 catalytic subunit	100%
3	Xaa-Pro dipeptidase	100%
4	Thimet oligopeptidase	100%
5	Aminopeptidase B	100%
6	Dipeptidyl peptidase 3	100%
7	Cathepsin D	100%
8	Bleomycin hydrolase	100%
9	72 kDa type IV collagenase	100%
10	Cytosolic non-specific dipeptidase	100%
11	Cathepsin Z	100%
12	Caspase-14	100%
13	Gamma-glutamylcyclotransferase	97%
14	Protein-glutamine gamma-glutamyltransferase 2	95%
15	Trypsin-3	95%

Supplemental Table 9. SiRNA sequences

1803-1 (Human, DPP4)	RNA-GAC ACU GUC UUC AGA CUG A=tt (1-AS)
1803-1 (Human, DPP4)	RNA-UCA GUC UGA GAG UGU C=tt(1-AA)
1803-2 (Human, DPP4)	RNA-CAG AUG AUA ACG ACU U=tt (2-AS)
1803-2 (Human, DPP4)	RNA-AAG UGA UUA UCA UCU G=tt (2-AA)
1803-3 (Human, DPP4)	RNA-UCU ACU CUG AUG AGU CAC U =tt (3-AS)
1803-3 (Human, DPP4)	RNA-AGU GAC UCA UCA GAG UAG A=tt (3-AA)
9520-1 (Human, PSA)	RNA-CAG CUU GUC UUU CCC UAG U=tt (4-AS)
9520-1 (Human, PSA)	RNA-ACU AGG GAA CAA GGU G=tt (4-AA)
9520-2 (Human, PSA)	RNA-GUC AUC AGA UUG UGA GAC U=tt (5-AS)
9520-2 (Human, PSA)	RNA-AGU CUC ACA AUC UGA UGA C=tt (5-AA)
9520-3 (Human, PSA)	RNA-GUC AGU GUG GGC CAU CCA U=tt (6-AS)
9520-3 (Human, PSA)	RNA-AUG GGC CCA CAC CAC UGA C=tt (6-AA)
(Bioneer Co. Daejeon, Republic of Korea).	

Supplemental Table 10. Preparation of siRNA reagents. Tube A and B were mixed and 20 µL was dropped into each well (siRNA final concentration: 30nM).

		DPP4	PSA	Control
**Tube A**	Opti-MEM	25 µL	25 µL	25 µL
	Lipofectamine® RNAiMAX	1.5 µL	1.5 µL	1.5 µL
**Tube B**	Opti-MEM	25 µL	25 µL	25 µL
	siRNA (30 µM)	0.5 µL	0.5 µL	0.5 µL

Supplemental Table 11. Sequences of primers used for quantitative PCR

DPP4	forward:	5’- AGTGGCGTGTTCAAGTGTGG -3’
	reverse:	5’- CAAGGTTGTCTTCTGGAGTTGG -3’
PSA	forward:	5’- CAGTTGAGGGATTTGCAGTTG-3’
	reverse:	5’- TGAAGGAGCTGGGTGACTCT -3’
GAPDH	forward:	5’- GAAGGTGAAGGTCGGAGTC-3’
	reverse:	5’- GAAGATGGTGATGGGATTTC-3’
HPRT1	forward:	5’- TTTGCTTTCCTTGGTCAGGC-3’
	reverse:	5’- GCTTGCGACCTTGACCATCT-3’

Supplemental Table 12. Reagents for quantitative PCR

Reagent	Per well
RNA-directTM SYBR®Green Realtime PCR Master Mix	10 µL
50 mM $Mn(OAc)_2$	1 µL
Forward Primer (50 µM)	0.1 µL
Reverse Primer (50 µM)	0.1 µL
Sterilized water (RNase-free)	6.8 µL
Nucleic acid extraction solution	2 µL
